# Pregnane X receptor (PXR) deficiency protects against spinal cord injury by activating NRF2/HO‐1 pathway

**DOI:** 10.1111/cns.14279

**Published:** 2023-06-02

**Authors:** Li‐Na Xuan, Zhen‐Xin Hu, Zhen‐Fu Jiang, Cong Zhang, Xiao‐Wan Sun, Wen‐Hua Ming, Hui‐Tao Liu, Rong‐Fang Qiao, Lin‐Jie Shen, Shao‐Bo Liu, Guan‐Yu Wang, Lin Wen, Zhi‐Lin Luan, Jian Yin

**Affiliations:** ^1^ Department of Neurosurgery the Second Affiliated Hospital of Dalian Medical University Dalian China; ^2^ Epileptic Center of Liaoning the Second Affiliated Hospital of Dalian Medical University Dalian China; ^3^ Department of Orthopedics The First Affiliated Hospital of Dalian Medical University Dalian China; ^4^ Advanced Institute for Medical Sciences Dalian Medical University Dalian China; ^5^ Department of Orthopedics Taizhou Hospital of Zhejiang Province Linhai China; ^6^ Department of Gastroenterology Ningbo First Hospital Ningbo China; ^7^ Dalian Key Laboratory for Nuclear Receptors in Major Metabolic Diseases Dalian China

**Keywords:** activation, gene knockout, oxidative stress, Pregnane X receptor (PXR), spinal cord injury (SCI)

## Abstract

**Introduction:**

As a devastating neurological disease, spinal cord injury (SCI) results in severe tissue loss and neurological dysfunction. Pregnane X receptor (PXR) is a ligand‐activated nuclear receptor with a major regulatory role in xenobiotic and endobiotic metabolism and recently has been implicated in the central nervous system. In the present study, we aimed to investigate the role and mechanism of PXR in SCI.

**Methods:**

The clip‐compressive SCI model was performed in male wild‐type C57BL/6 (PXR^+/+^) and PXR‐knockout (PXR^−/−^) mice. The N2a H_2_O_2_‐induced injury model mimicked the pathological process of SCI in vitro. Pregnenolone 16α‐carbonitrile (PCN), a mouse‐specific PXR agonist, was used to activate PXR in vivo and in vitro. The siRNA was applied to knock down the PXR expression in vitro. Transcriptome sequencing analysis was performed to discover the relevant mechanism, and the NRF2 inhibitor ML385 was used to validate the involvement of PXR in influencing the NRF2/HO‐1 pathway in the SCI process.

**Results:**

The expression of PXR decreased after SCI and reached a minimum on the third day. In vivo, PXR knockout significantly improved the motor function of mice after SCI, meanwhile, inhibited apoptosis, inflammation, and oxidative stress induced by SCI. On the contrary, activation of PXR by PCN negatively influenced the recovery of SCI. Mechanistically, transcriptome sequencing analysis revealed that PXR activation downregulated the mRNA level of heme oxygenase‐1 (HO‐1) after SCI. We further verified that PXR deficiency activated the NRF2/HO‐1 pathway and PXR activation inhibited this pathway in vitro.

**Conclusion:**

PXR is involved in the recovery of motor function after SCI by regulating NRF2/HO‐1 pathway.

## Graphical abstract

PXR deficiency promoted the recovery of motor function after spinal cord injury (SCI) by inhibiting apoptosis, inflammation, and oxidative stress via NRF2/HO‐1 pathway. In contrast, pharmacological activation of PXR had a negative influence on SCI recovery. Therefore, as a novel therapeutic target for SCI, antagonism of PXR may have clinical potential for the rehabilitation therapy of SCI.

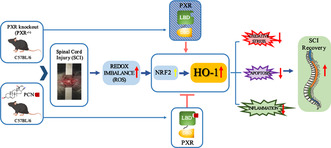

## 1 INTRODUCTION

Spinal cord injury (SCI) is a destructive central nervous system (CNS) trauma which not only imposes high physical, psychological, and occupational effects on the individual, but also causes a huge economic burden to the family and society.^1^, ^2^ Depending on the location of the injury and the amount of preserved spinal cord tissue, the clinical manifestations of SCI may include the partial or complete loss of movement, sensation, and control of bowel or bladder. The estimated global prevalence of SCI ranges from 236 to 1298 individuals per million.^3^ The therapies of SCI mainly include surgeries, drugs, and stem cell transplantation.^4^, ^5^ However, clinical improvements in patients are not satisfactory due to the unique pathological mechanism of SCI characterized by an initial primary injury followed by a progressive secondary injury. In general, the primary injury caused by direct physical forces leads to blood–spinal cord barrier disruption, hemorrhage, ischemia, axon disruption, and inflammatory cell infiltration. In contrast, secondary injury including the release of oxygen free radicals, astrogliosis, vessel thrombosis, and neuronal apoptosis, is an indirect result of primary injury resulting in sustained and extensive tissue damage.^2^ Among these mechanisms, oxidative stress is considered a critical event for the secondary injury progression.^6^, ^7^, ^8^ Oxidative stress is defined as an imbalance between oxidants and the organism's capacity to counteract the action of antioxidants, in which the overproduction of free radicals is a key process.^9^, ^10^, ^11^ Oxidative stress can damage the function of lipids, nucleic acids, and proteins. Thus, lipids peroxidation yields malondialdehyde (MDA) and 4‐hydroxynonenal (4‐HNE), nucleic acids peroxidation yields 8‐hydroxydeoxyguanosine (8‐OXO), and proteins peroxidation inactivate proteins. Due to the fact that the spinal cord has a high rate of oxidative metabolic activity and is rich in polyunsaturated n‐3 fatty acids,^12^ spinal cord is particularly prone to peroxidation. Previous studies have shown that free radicals are significantly increased after SCI.^13^, ^14^ Therefore, the prevention of oxidative stress development may be an effective way to alleviate SCI.

The members of nuclear receptors (NR) superfamily are ligand‐dependent transcription factors that interact with co‐regulatory factors to regulate the expression of genes linked to cell development, detoxification, metabolism, inflammation, and reproduction.^15^, ^16^ So far, 49 NRs were found in mouse, 47 in the rat, and 48 in human.^17^ Nearly, all NRs share the same domain structure including^18^, ^19^: a highly disordered N‐terminal domain (NTD) containing a ligand‐independent transcription region called activation function‐1 (AF‐1); a conserved DNA‐binding domain (DBD) residing approximately along the center; a ligand‐binding domain (LBD) positioning along the C‐terminal and containing a ligand‐dependent activator function‐2 (AF‐2) domain; DBD and LBD are linked by hinge region keeping the structural flexibility. Ligand binding induces conformational changes in the receptor and recruitment of co‐regulators to complete gene expression activation or inhibition.^20^ Increasing evidence suggests that NRs play an important role in nervous system‐related diseases including SCI,^21^ Alzheimer's disease (AD),^22^, ^23^, ^24^ Parkinson's disease (PD),^25^, ^26^ Niemann‐Pick type C1 (NPC1) disease,^27^ and amyotrophic lateral sclerosis (ALS).^28^

Nuclear receptor subfamily 1 group I member 2 (NR1I2) was discovered in 1998 and found first to respond to endogenous pregnanes so it was also named pregnane X receptor (PXR).^29^ Subsequently, PXR was found to be activated by both endogenous and exogenous compounds to regulate phase I and phase II enzymes and drug transporters. Therefore, PXR has been defined as a xenobiotic receptor. PXR is enriched in metabolic tissues such as liver and gastrointestinal tract and also expressed moderately in other organs including kidneys, brain, and heart.^30^ The role of PXR is further extended by recent evidence indicating PXR signaling in inflammation, apoptosis, proliferation, and oxidative stress,^31^, ^32^, ^33^ which are closely related to the physiological and pathological processes of CNS. Therefore, increasing attention has been paid to the role of PXR in CNS. Adult C57BL/6 J PXR^−/−^ mice developed anxiety‐like behavior and recognition memory impairment.^34^ Moreover, the knockdown of PXR reduced nonylphenol‐induced apoptosis and neurotoxicity in mouse hippocampal cells.^35^ In NPC1 disease, the neurosteroid allopregnanolone (ALLO) can improve survival and neurological function in *npc1* ^−/−^ mice by activating PXR.^27^ Compared with normal people, the expression of PXR was increased in the brain microvascular endothelial cells of patients with drug‐resistant epilepsy. PXR led to drug resistance by promoting the expression of drug metabolism enzymes and drug transporters.^36^ Nevertheless, the functions and mechanisms of PXR in the SCI remain poorly understood. In the present study, we aimed to investigate the role and mechanism of PXR in SCI development by manipulating the expression and activation of PXR in vivo and in vitro.

## 2 MATERIALS AND METHODS

### 2.1 Animals

Adult male C57BL/6 mice (8–10 weeks) were purchased from Beijing HFK Bioscience Co., Ltd. The PXR gene‐knockout (PXR^−/−^) mice were generated as previously described,^37^ genotyped, and confirmed as shown in Figure S1. Homozygous wild‐type (PXR^+/+^) mice and gene‐knockout (PXR^−/−^) mice were generated from inbred heterozygous PXR^+/−^ mice. Animals were maintained under standard animal care conditions (12‐h light/dark cycle, room temperature 23°C) with access to food and water ad libitum in the animal facility of Dalian Medical University. All the animal experiments were approved by the Laboratory Animal Center, Dalian Medical University.

### 2.2 Surgical procedures and treatment

The clip‐compressive SCI model was described in previous studies.^38^, ^39^ Briefly, mice were anesthetized (20 mL/kg) intraperitoneally with avertin (2, 2, 2‐tribromoethanol, Macklin, Shanghai, China) in 0.9% saline. A laminectomy from T8 to T10 was performed with a surgical microscope (Nikon, Tokyo, Japan). Spinal cord was subjected to the compression of a vascular clip (30 g) for 10 s in T9.^39^, ^40^ After surgery, the animals' bladders were manually evacuated twice daily until the restoration of normal bladder function. Mice in the sham group were subjected to laminectomy alone. All animals were male in view of pregnane having the ability to activate PXR. To evaluate the effect of the *PXR* gene on SCI, 8–10 weeks old C57BL/6 wild‐type (PXR^+/+^) mice and PXR gene‐knockout (PXR^−/−^) mice were divided into 4 groups (*n* = 6–8 per group): Sham/PXR^+/+^, Sham/ PXR^−/−^, SCI/PXR^+/+^, and SCI/ PXR^−/−^. To assess the role of the mouse PXR agonist PCN (pregnenolone 16α‐carbonitrile, Sigma‐Aldrich, Beijing, China) on SCI, 8–10 weeks old C57BL/6 wild‐type mice were randomly divided into 4 groups (*n* = 6–8 per group): Sham/Oil, Sham/PCN, SCI/ Oil, and SCI/PCN. Mice were pretreated via oral gavage daily with PCN (40 mg/kg) or the same volume of corn oil for 3 days. The mice were then subjected to SCI surgery and continued with daily PCN (40 mg/kg) or the same volume of corn oil for 7 days. All animals were used to assess histological, biochemical, and motor function.

### 2.3 Locomotion recovery assessment

Behavioral analyses were performed by two well‐trained observers who were unaware of genotypes and treatments.

#### 2.3.1 Basso mouse scale (BMS) scoring analysis

BMS open field test is a 9‐point scale, from 0 point (no hind limb movement) to 9 points (coordinated gait), for detecting the recovery of hind limbs motor function in mice. Mice were walked freely in an open field, and hind limbs movement was observed for 5 min pre‐operatively and on days 1, 3, and then once a week after the surgery.

#### 2.3.2 Inclined plane test

The inclined plane test was used to evaluate the recovery of hind limbs' function.^41^ Briefly, mice were placed on an inclined rubber plane with increasing angle, then record the maximum angle at which the mice could hold their position for more than 5 s without falling.

#### 2.3.3 Footprint analysis

After dyeing hind paws in red and forepaws in black, the mice were put to run along a suitable box (3 feet long, 2 inches wide) with paper‐lined.^42^

### 2.4 Tissue preparation

Spinal cord samples were obtained before surgery and at 1, 3, 7, 14, 21, and 28 days after surgery. After perfusing the mice with 0.9% NaCl followed by 4% paraformaldehyde (PFA, Solarbio, Beijing, China), 1 cm spinal cords in length taken from the central area of the injury were immersed in 4% PFA overnight (18–24 h) and transferred to 30% sucrose solution until tissues sank to the bottom, then spinal cords were embedded in paraffin. Longitudinal sections of spinal cords (10 μm thick) were processed for staining.

### 2.5 Section preparation for histopathological staining

Paraffin sections were heated at 60°C for 30 min, then placed in xylene I and II for 20 min each, followed by gradient alcohol solutions of 100%, 95%,85%, and 75% for 5 min each, and finally were washed with steamed water. These treatments were used to prepare the tissues for all staining.

### 2.6 Hematoxylin–eosin (HE) staining

After staining with hematoxylin for 3–5 min, the sections were treated with double distilled water, differentiation solution, double distilled water, blue returning solution, and running tap water. The sections were then stained with eosin for 5 min, dehydrated by 95% ethanol, 100% ethanol, cleared in xylene, and finally covered by neutral resins. Images were taken with an upright optical microscope (Nikon, Tokyo, Japan).

### 2.7 Nissl staining

After the sections were incubated with Nissl staining solution (Beyotime Institute of Biotechnology, Shanghai, China) for 20–40 min at room temperature, the sections were rinsed in double distilled water, dehydrated by different concentrations of ethanol, cleared in xylene, and finally covered with neutral resins. Images were acquired using an upright optical microscope (Nikon, Tokyo, Japan).

### 2.8 Immunohistochemical staining

The sections were blocked in 3% H_2_O_2_ for 10 min to inhibit endogenous peroxidase activity followed by incubation in 3% BSA blocking buffer for 30 min. Thereafter, the sections were incubated with F4/80 polyclonal antibody (1:200, 28,463‐1‐AP, Proteintech, Wuhan, China) overnight, at 4°C, and rinsed with PBST. After using the DAB to produce a brown color, the nuclei were counterstained with hematoxylin. Images were acquired by using an upright optical microscope (Nikon, Tokyo, Japan). Three slices for each group were used for statistical analysis, and two fields were selected on each slice for obtaining signaling data.

### 2.9 Immunofluorescent staining

After blocking in 3% BSA at 37°C for 1 h, the sections were overnight incubated at 4°C with the following antibodies: anti‐PXR (1:100, ab192579, Abcam, Cambridge, UK), anti‐4‐hydroxynonenal (4‐HNE, 1:50, ab46545, Abcam, Cambridge, UK), anti‐8‐oxo‐2′ ‐deoxyguanosine (8‐OXO, 1:50, ab206461, Abcam, Cambridge, UK), anti‐NLRP3 (1:200, BA3677, Boster, Wuhan, China), anti‐NeuN (1:500, MAB377, Millipore, MA, USA), anti‐GFAP (1:500, MAB360, Millipore, MA, USA), anti‐Iba‐1 (1:500, GB11105, Servicebio, Wuhan, China). They were subsequently washed with PBST and added with drops of appropriate fluorescence‐conjugated secondary antibodies (1:1000, Invitrogen, MA, USA) at room temperature for 1 h. The nuclei were stained with DAPI (Solarbio, Beijing, China) at room temperature for 20 min. Images were taken with a fluorescent microscope (Nikon, Tokyo, Japan). Three slices for each group were used for statistical analysis, and two fields were selected on each slice for abstaining signaling data.

### 2.10 TUNEL staining

The sections were stained by using a terminal deoxynucleotidyl transferase dUTP nick‐end labeling (TUNEL) kit (11,684,817,910, Roche, Shanghai, China) to detect apoptotic cells. Briefly, after incubating with proteinase K for 15 min at 37°C, sections were covered with the TUNEL reaction mixture for 1 h under a humid atmosphere at 37°C. Finally, the sections were stained with DAPI at room temperature for 20 min to visualize the nucleus. The fluorescence images were obtained using a fluorescence microscope (Nikon, Tokyo, Japan).

### 2.11 Measurement of MDA content, SOD activity, and GPx activity

On day 3 postoperatively, the spinal tissues were taken from the central area of the injury (T9, 1 cm). N2a mouse neuroblast cells with different treatments were extracted. The samples were homogenized with a particular lysis buffer and the supernatant was collected for testing. The MDA Content Detection Kit (E‐BC‐K028‐M, Elabscience, Wuhan, China), SOD Activity Detection Kit (E‐BC‐K020‐M, Elabscience, Wuhan, China), and GPx Activity Detection Kit (S0059S, Beyotime Institute of Biotechnology, Shanghai, China) were used to detect MDA content, superoxide dismutase (SOD) activity, and glutathione peroxidase (GPx) activity according to the instructions of the manufacturers.

### 2.12 Western blot analysis

Spinal cords and N2a cells were lysed in the lysis buffer (20–188, Millipore, MA, USA) with the protease inhibitor cocktail (K1007, APEXBIO, Houston, USA), and incubated at 4°C for 30 min. Then the supernatants were collected after being centrifuged at 2,1130 *g* for 15 min at 4°C. The protein content was measured using the bicinchoninic acid assay kit (Sigma–Aldrich, Beijing, China), and mixed with 5× loading buffer (P0015L, Beyotime Institute of Biotechnology, Shanghai, China) to denature for 10 min at 95°C. The denatured samples were separated using 10% or 12% SDS‐polyacrylamide gel electrophoresis (SDS‐PAGE) and transferred onto polyvinylidene difluoride (PVDF) membranes (Millipore, MA, USA). After blocking in 5% skim milk (232,100, BD Bioscience, NJ, USA) at 25°C for 1.5 h, membranes were incubated with different primary antibodies overnight at 4°C. Primary antibodies included anti‐PXR (1:1000, ab192579, Abcam, Cambridge, UK), anti‐GAPDH (1:5000, 60,004‐1‐Ig, Proteintech, Wuhan, China), anti‐BAX (1:1000, ab182734, Abcam, Cambridge, UK), anti‐BCL2 (1:1000, 3498S, CST, MA, USA), anti‐NRF2 (1:1000, 16,396‐1‐AP, Proteintech, Wuhan, China), and anti‐HO‐1 (1:1000, 10,701‐1‐AP, Proteintech, Wuhan, China). After being washed thrice, the membranes were incubated for 1 h at room temperature with horseradish peroxidase (HRP)‐conjugated anti‐rabbit or anti‐mouse (ABclonal Technology, MA, USA). After being washed three times, the western blots were detected by the ECL detection kit (SQ201, Shanghai Epizyme Biomedical Technology Co., Ltd, Shanghai, China), and Tanon 5200 ECL detection system (YPH‐Bio, Beijing, China) was used to visualize immunoreactive bands.

### 2.13 Quantitative real‐time PCR (qRT‐PCR)

Total RNA was extracted from the spinal cord tissues of mice and N2a cells following homogenization in Trizol (R401‐01, Vazyme Biotech, Nanjing, China) according to the manufacturer's instructions. RNA was then reversely transcribed into cDNA with a reverse transcription kit (Q711‐01, Tiangen, Beijing, China). The expression level of PXR mRNA was quantified using ChamQ Universal SYBR qPCR Master Mix (Q711‐02, Vazyme Biotech, Nanjing, China) on the Real‐Time PCR System (Thermo Fisher, MA, USA). PXR primer: forward, 5′‐ GGT GTG GTC CAG CGC AGC GT‐3**′** and reverse, 3**’**‐ACT GCT GGG TTT GCT GGG CGT‐5′^37^; GAPDH primer: forward, 5′‐ AAG AAG GTG GTG AAG CAG G‐3′; reverse, 3**′**‐GAA GGT GGA AGA GTG GGA GT‐5′.

### 2.14 RNA sequencing and analysis

Mice were treated as follows: Sham/Oil, Sham/PCN, SCI/Oil, SCI/PCN. On the third day after surgery, the mice were anesthetized and perfused with 0.9% NaCl to take the lesioned spinal cords (T9, 1 cm). Total RNA was extracted following homogenization in Trizol. After using the RNA Nano 6000 Assay Kit of the Bioanalyzer 2100 system (Agilent Technologies, CA, USA) to check the integrity of the RNA, only when the RNA integrity number (RIN) was >8, the samples were used for the establishment of a transcriptome library. The sequencing and analysis were provided by the Novogene Co, Ltd, (Beijing, China).

### 2.15 Cell viability assay

Cell viability was assessed by Cell Counting Kit‐8 Kit (CCK‐8, APEXBIO, Houston, USA). In brief, cells were seeded on 96‐well plates and incubated at 37°C, 5% CO_2_ for 24 h, then exposed to different treatments. At the end of each experiment, the culture solution was discarded and 110 μL of Dulbecco's Modified Eagle's Medium (DMEM, Gibco, Paisley, UK) containing 10 μL CCK‐8 was added at 37°C for 2 h each incubation. The optical density (OD) 450 nm values were measured using the multimode microplate reader (TECAN, Männedorf, Switzerland).

### 2.16 Cell culture and treatment of H_2_O_2_

N2a cells were seeded into dishes in DMEM containing 10% fetal bovine serum (FBS, Thermo Fisher, MA, USA) with 100 μg/mL penicillin and 100 μg/mL streptomycin (Beyotime Institute of Biotechnology, Shanghai, China) in a humid atmosphere of 5% CO_2_ at 37°C. Media was renewed on alternative days. To induce oxidative stress condition in vitro, cells were treated with 0, 100, 200, 300, 400, and 500 μM H_2_O_2_ for 24 h, and cell viability was analyzed using CCK‐8 to assess the effect of cytotoxic influence. According to inhibitory concentration (IC50) of H_2_O_2_, 400 μM H_2_O_2_ was used for all further experiments.

### 2.17 Cell transfection

The siRNA targeting PXR (sense 5′‐ GCG ACG GGA AAG AGA UCA UTT‐3′ and anti‐sense 5′‐AUG AUC UCU UUC CCG UCG CTT‐3′) and the negative control were obtained from Genepharma (Shanghai, China). N2a cells were transiently transfected with siPXR (50 nM) using Lipofectamine 3000 (Thermo Fisher, CA, USA) according to the instructions provided by the manufacturer, and the negative control of siRNA was used as control. After 36 h, the transfected cells were exposed to 400 μM H_2_O_2_ for 24 h.

### 2.18 Treatment of ML385 and PCN

The NRF2 inhibitor (ML385) was purchased from Medchem Express (#HY‐100523; MCE Co. Ltd., Shanghai, China). The dose of ML385 (5 μM) was determined according to previous studies.^43^, ^44^ N2a cells were pretreated with ML385 (5 μM) for 12 h, followed by exposure to H_2_O_2_ (400 μM) for 24 h.

PCN was dissolved in DMSO as a stock solution and then diluted into 40 μM working concentration. N2a cells were treated as follows: DMSO, PCN 6 h, H_2_O_2_/DMSO, H_2_O_2_/PCN 6 h, H_2_O_2_/PCN 30 h. Briefly, N2a cells were maintained in DMEM with 10% FBS to reach 50% confluence, then replaced by DMEM containing PCN as the pretreatment for 6 h to PCN 6 h, H_2_O_2_/PCN 6 h and H_2_O_2_/PCN 30 h, and DMSO only were used as controls to other all groups. Subsequently, cells were exposed to 400 μM H_2_O_2_ for 24 h to H_2_O_2_/DMSO, H_2_O_2_/PCN 6 h, and H_2_O_2_/PCN 30 h. At the same time, PCN was continuously stimulated for 24 h to H_2_O_2_/PCN 30 h. Cells were not treated with H_2_O_2_ to DMSO and PCN 6 h groups.

### 2.19 Cultured cell immunofluorescence staining

For cell immunofluorescence staining, the protocols were as previously described.^45^ In brief, N2a cells cultured on glass chamber slides were rinsed once with PBS and fixed in 4% PFA at room temperature for 20 min. Then cells were blocked and permeabilized with 0.1% Triton X‐100 in PBS containing 3% BSA at room temperature for 1 h. Subsequently, they were incubated overnight with anti‐4‐hydroxynonenal (4‐HNE, 1:50, ab46545, Abcam, Cambridge, UK) at 4°C. Slides were washed with PBS and incubated with fluorescence‐conjugated secondary antibodies (1:1000, Invitrogen, MA, USA) at room temperature for 1 h. After washing with PBS, the sections were stained with DAPI (Solarbio, Beijing, China) at room temperature for 10 min. Images were acquired by using a fluorescent microscope (Nikon, Tokyo, Japan).

### 2.20 Statistics

Data were analyzed using GraphPad Prism 8.0 (GraphPad Software, Inc., LA Jolla, CA, USA). The significance of differences was performed by unpaired *t*‐test or one‐way ANOVA with statistical significance set at a *p*‐value of <0.05. All results were given as arithmetic means ± standard error of the mean (SEM). A detailed description of significance levels was given in the figure legends.

## 3 RESULTS

### 3.1 PXR is specifically expressed in neurons of spinal cord

**FIGURE 1:**
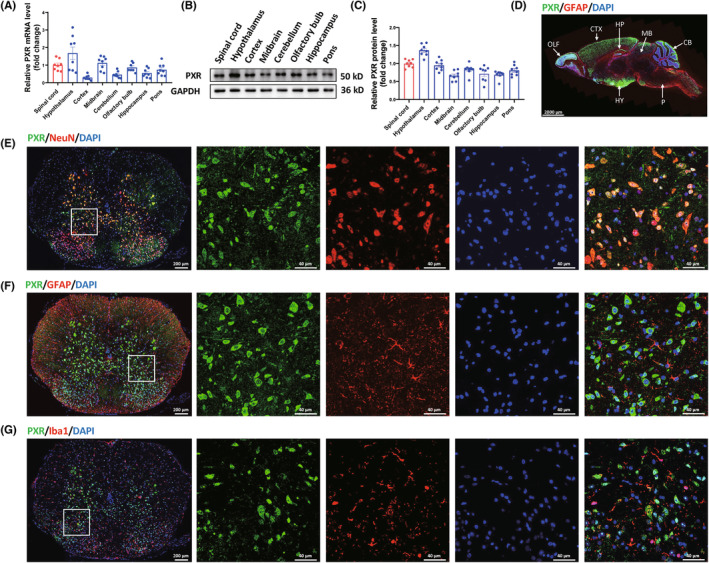
PXR expression pattern in mouse nervous system. (A) Real‐time PCR analysis showed the relative mRNA level of *PXR* in different regions of mouse nervous system (*n* = 8 per brain region). (B) Western blot analysis showed the protein expression of PXR in different regions of mouse nervous system. (C) Quantitative analysis of PXR protein level in different regions of mouse nervous system as shown in (B) (*n* = 8 per brain region). (D) Immunofluorescent staining showed the expression pattern of PXR in sagittal section of mouse brain; hypothalamus (HY), cerebral cortex (CTX), midbrain (MB), cerebellum (CB), olfactory bulb (OLF), hippocampus (HP), pons (P). (E) Colocalization of PXR with neurons in mouse spinal cord was detected by double immunofluorescent staining of PXR (green) and NeuN (red). (F) Colocalization of PXR with astrocytes in mouse spinal cord was detected by double immunofluorescent staining of PXR (green) and GFAP (red). (G) Colocalization of PXR with microglia in mouse spinal cord was detected by double immunofluorescent staining of PXR (green) and Iba1 (red).

To determine the expression pattern of PXR in the mouse CNS, real‐time PCR was used to measure the PXR mRNA expression level, and western blot analysis and immunofluorescence were applied to examine the PXR protein level in various regions of CNS. The results showed that PXR was widely expressed in CNS with the highest expression in the hypothalamus, followed by the cerebral cortex and spinal cord (Figure 1A–D). It has been previously reported that PXR is expressed in neurons and vascular endothelium of human drug‐resistant epileptic brains.^36^ We also examined the PXR expression in a human brain tissue specimen from a patient with intracranial hematoma, which showed that PXR existed in neurons confirmed by its co‐localization with NeuN, a neuron‐specific marker (Figure S2). We further investigated the spatial expression pattern of PXR in spinal cord, we performed double immunofluorescent staining of PXR and neural cell markers, including NeuN, GFAP (an astrocyte‐specific marker), and Iba1 (a microglia‐specific marker) in the adult male mouse spinal cords. The staining results showed that PXR was only expressed in NeuN^+^ neurons, but not in GFAP^+^ astrocytes and Iba1^+^ microglia (Figure 1E–G). We also validated the knockout of PXR in spinal cord of PXR^−/−^ mice by double immunofluorescent staining (Figure S3).

### 3.2 PXR expression is regulated after SCI in mice

**FIGURE 2:**
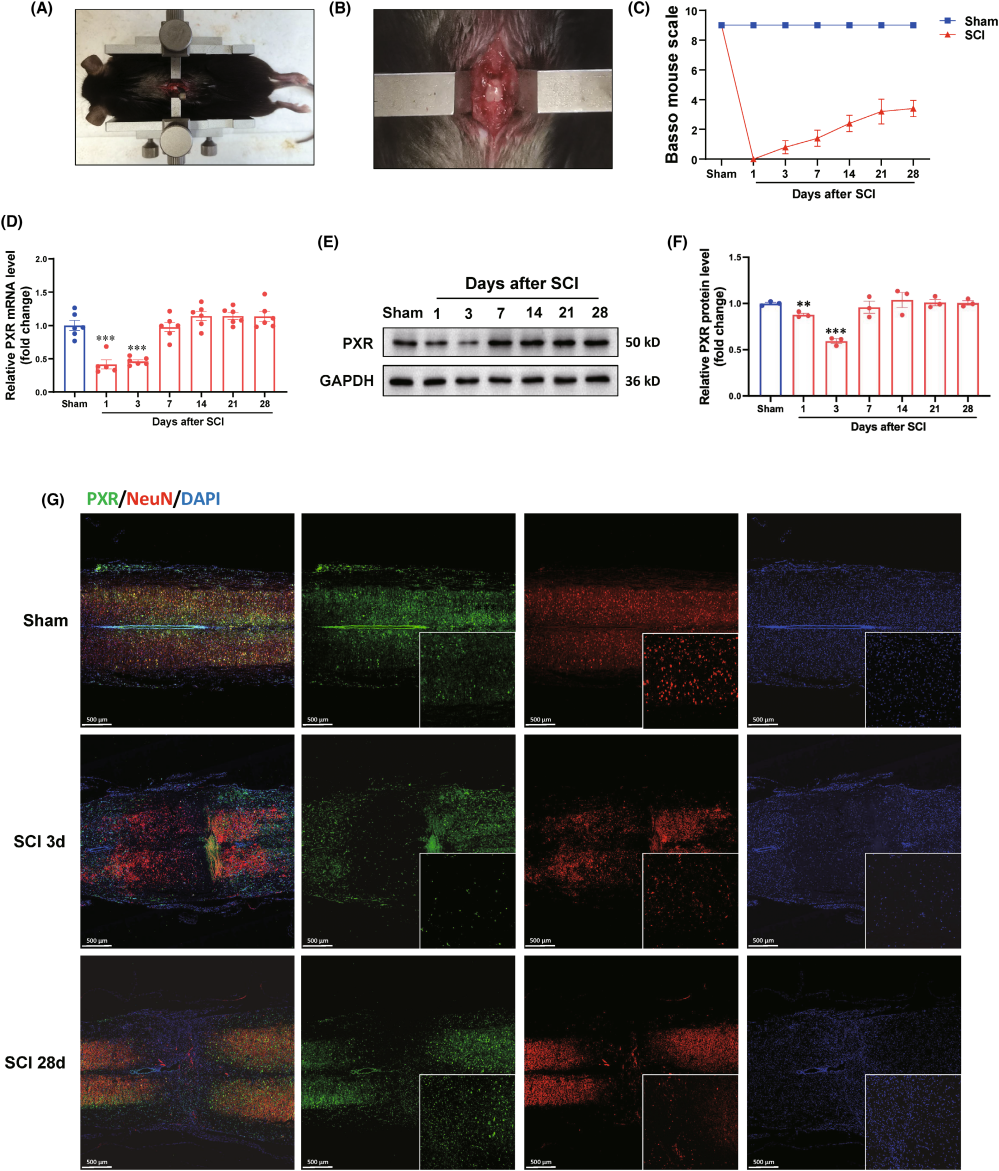
Regulated PXR expression after spinal cord injury in mice. (A,B) Typical images showed the construction of clip‐compressive SCI model. (C) Quantitative analysis of gross voluntary movement measured by BMS scoring analysis at different stages after SCI (*n* = 5 per group). (D) Real‐time PCR analysis showed the relative mRNA level of PXR at different stages after SCI (*n* = 6 per group). (E) Western blot analysis showed the protein expression of PXR at different stages after SCI. (F) Quantitative analysis of protein level at different stages after SCI is shown in (E) (*n* = 3 per group). (G) PXR expression was detected by double immunofluorescent staining of PXR (green) and NeuN (red) at different stages after SCI. **p ˂ 0.01, ***p ˂ 0.001 compared with the Sham group. Data were presented as mean ± SEM.

To explore the effects of PXR on SCI, the clip‐compressive SCI model was performed. The moderate–severe crush injury was made by bilateral compression using a vascular clip which caused a clear injury zone in T9 of spinal cord and paralysis of the hind feet (Figure 2A–C). The pathological characteristics of SCI were examined by HE staining and typical Nissl staining (Figure S4). Based on this SCI model, we then examined the mRNA expression pattern of PXR in different stages of SCI by real‐time PCR. We found that the mRNA of PXR declined and reached a minimum between 1 and 3 days at the injury site after SCI (Figure 2D). Furthermore, we used western blot analysis (Figure 2E,F) and immunofluorescence (Figure 2G and Figure S5) to examine the temporal and spatial expression pattern of PXR. Our data showed that PXR was localized in the NeuN^+^ neurons, and the PXR protein level was significantly decreased at the lesion site, with the minimum level at 3 days, and return to basal level at 7–28 days after SCI.

### 3.3 PXR knockout ameliorates the recovery of motor function and histopathological damage after SCI

**FIGURE 3:**
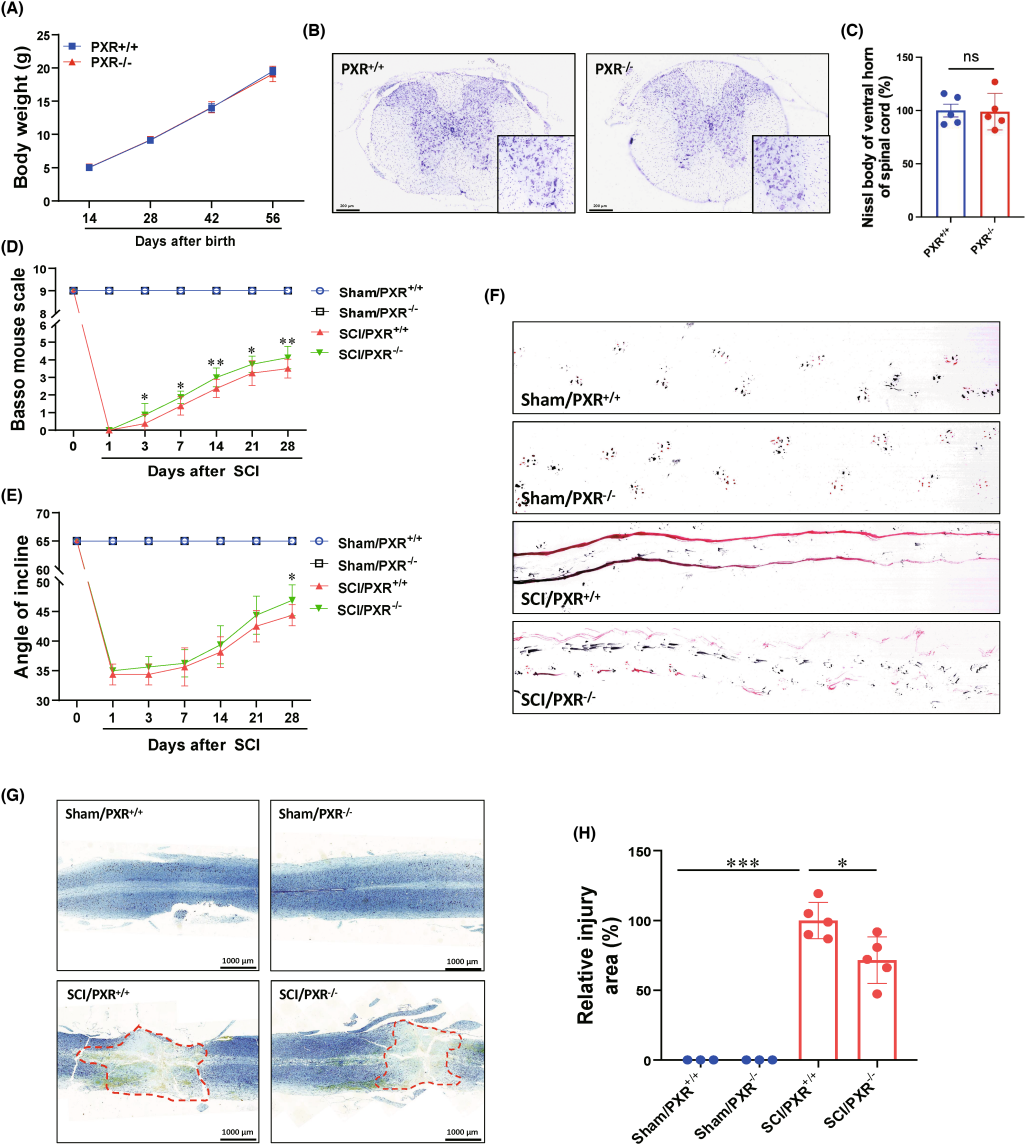
PXR knockout protects against spinal cord injury in mice. (A) Body weight of wild‐type (PXR^+/+^) and PXR knockout (PXR^−/−^) mice (*n* = 5 per group). (B) Nissl staining images showed the nissl bodies in the ventral horn of the spinal cords of PXR^+/+^ and PXR^−/−^ mice. (C) Quantitative analysis of nissl bodies in (B). (D,E) Quantitative analysis of gross voluntary movement of PXR^+/+^ and PXR^−/−^ mice measured by BMS scoring analysis (D) and inclined plane test (E) at different stages after SCI (*n* = 8 per group). (F) Representative footprint images of PXR^+/+^ and PXR^−/−^ mice after SCI. (G) Nissl staining images showed the injury area in the spinal cords of PXR^+/+^ and PXR^−/−^ mice after SCI. (H) Quantitative analysis of the injury area in the spinal cord is shown in (G). *p ˂ 0.05, **p ˂ 0.01, ***p ˂ 0.001. Data were presented as mean ± SEM.

Due to the decreased expression of PXR in neurons at 3 days after SCI, we hypothesized that it may play a critical role in neurological function recovery. PXR^−/−^ mice were bred to demonstrate the role of PXR in SCI (Figure S1). We found that there was no obvious difference in the body weight, neuronal number in the spinal cords, and motor function between PXR^+/+^ and PXR^−/−^ mice (Figure 3A–F). Further, PXR^+/+^ and PXR^−/−^ mice were subjected to SCI before being observed the motor function at 1, 3, 7, 14, and 28 days. The results showed that the BMS motor scores declined to 0 at 1 day in all SCI groups, and motor scores gradually increased with the time of injury. Compared to PXR^+/+^ mice, PXR^−/−^ mice consistently showed higher motor function scores from 3 days after SCI (Figure 3D). In addition, we found that PXR^−/−^ mice showed better performance of hind limb motor function at 28 days after SCI by inclined plane test and footprint behavioral assays (Figure 3E,F). Longitudinal HE staining and Nissl staining were performed on spinal cord samples in the Sham/PXR^+/+^, Sham/PXR^−/−^, SCI/PXR^+/+^, and SCI/PXR^−/−^ groups on 28 days after the operation (Figure S6, Figure 3G). Compared with PXR^+/+^ mice, PXR^−/−^ mice showed smaller injury size after SCI (Figure 3G,H). Taken together, these results suggested that deletion of the *PXR* gene promoted the recovery of motor function.

### 3.4 PXR deficiency alleviates apoptosis and inflammation induced by SCI

**FIGURE 4:**
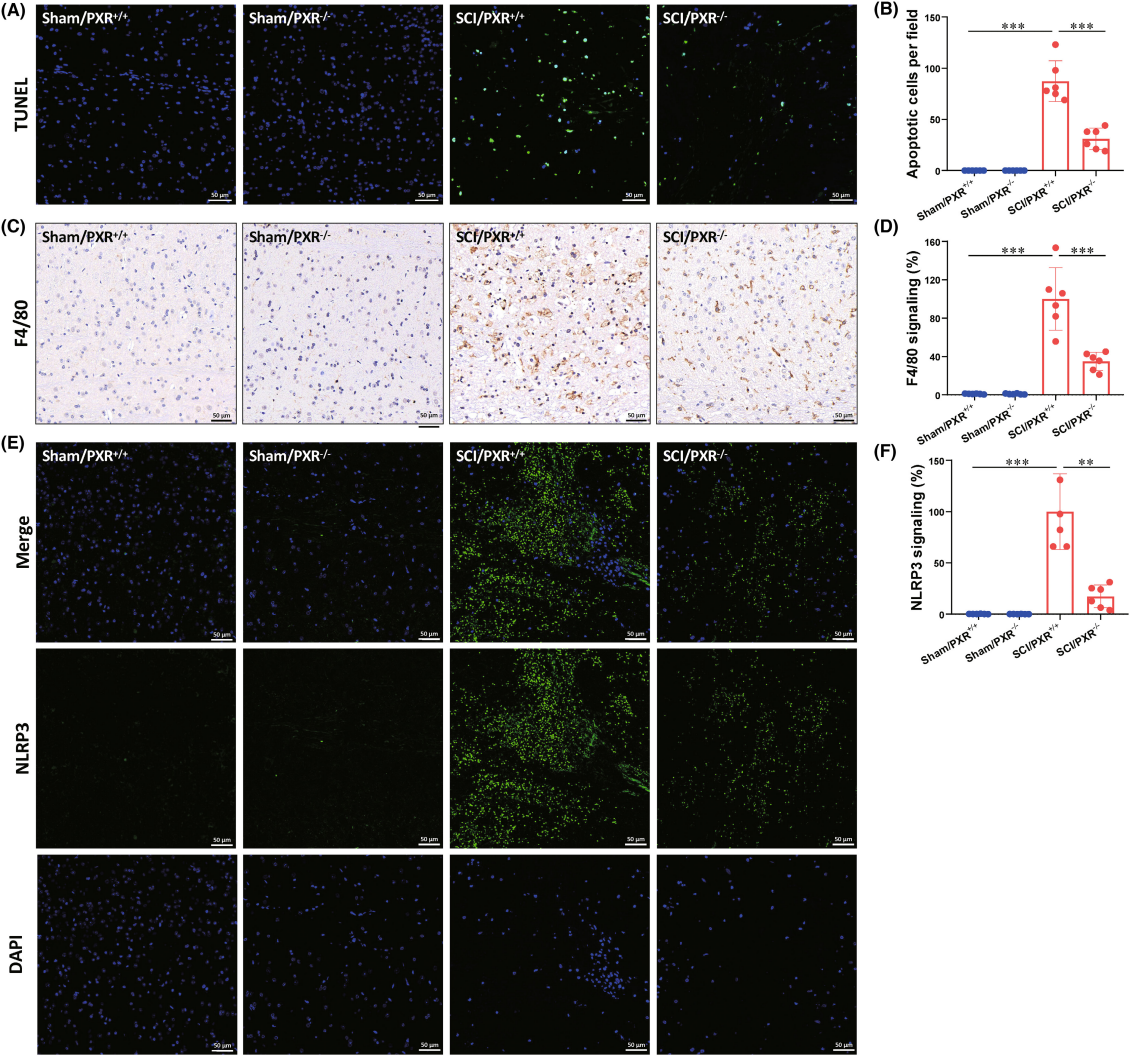
PXR deficiency alleviates apoptosis and inflammation induced by spinal cord injury. (A) Apoptotic cells were detected by TUNEL staining after SCI in PXR^+/+^ and PXR^−/−^ mice. (B) Quantitative analysis of apoptosis in (A) (*n* = 6 per group). (C) Immunohistochemical staining showed the F4/80 expression after SCI in PXR^+/+^ and PXR^−/−^ mice. (D) Quantitative analysis of F4/80 signaling in (C) (*n* = 6 per group). (E) Immunofluorescent staining showed the NLRP3 expression after SCI in PXR^+/+^ and PXR^−/−^ mice. (F) Quantitative analysis of NLRP3 signaling in (E) (*n* = 6 per group). **p ˂ 0.01, ***p ˂ 0.001. Data were presented as mean ± SEM.

Previous studies have shown that inhibiting the progression of secondary injury in SCI, such as apoptosis and inflammation, contributes to the acceleration of SCI recovery.^46^, ^47^ As PXR expression reached its minimum at 3 days after SCI, we selected tissues at 3 days after SCI for mechanistic studies. TUNEL staining was used to analyze the number of apoptotic cells in each group. We found significantly increased apoptosis in SCI/PXR^+/+^ group compared to Sham/PXR^+/+^ group, which was alleviated in SCI/PXR^−/−^ group (Figure 4A,B). Immunohistochemical staining showed that a microglia marker F4/80 was remarkably expressed in SCI/PXR^+/+^ group. However, the expression of F4/80 was significantly decreased in SCI/PXR^−/−^ group compared to SCI/PXR^+/+^ group (Figure 4C,D). Since the formation of inflammasomes is considered as the key factor in the initiation of neuroinflammation and NOD‐like receptor protein 3 (NLRP3) is a key member of the inflammasome in SCI.^48^ Therefore, we performed immunofluorescence analysis to examine the expression of NLRP3 after SCI. Compared to the SCI/PXR^+/+^ group, NLRP3 expression was remarkably suppressed in SCI/PXR^−/−^ group (Figure 4E,F). Together, these data demonstrated that PXR deficiency alleviated apoptosis and inflammation during the early stage of SCI.

### 3.5 PXR deficiency inhibits oxidative stress induced by SCI

**FIGURE 5:**
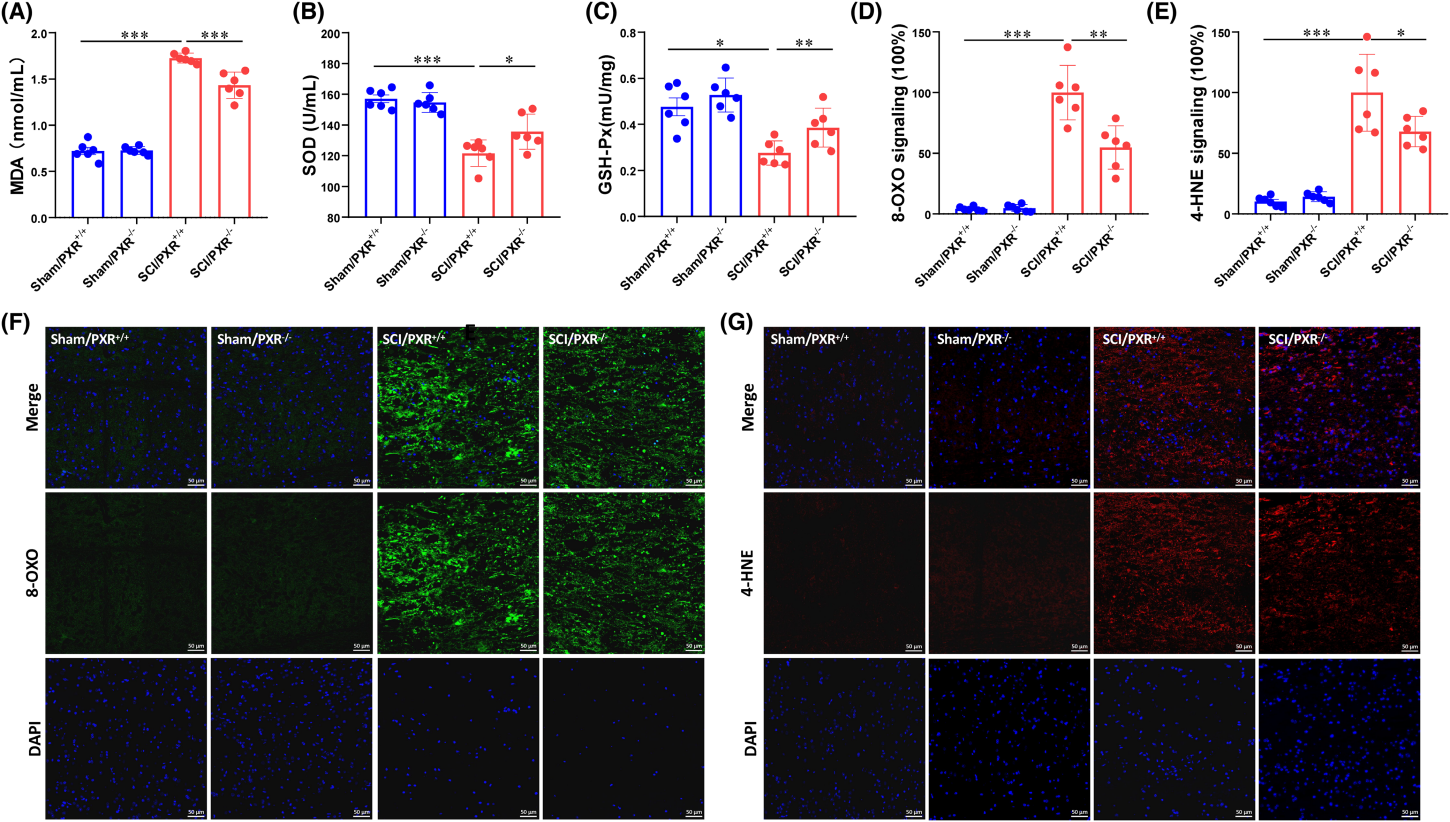
PXR deficiency inhibits oxidative stress induced by spinal cord injury. (A–C) Biochemical indexes, MDA content (A), SOD activity (B), and GPx activity (C), were measured in the spinal cord tissues of PXR^+/+^ and PXR^−/−^ mice after SCI (*n* = 6 per group). (D,F) Immunofluorescent staining and corresponding quantitative analysis showed the 8‐OXO expression in the spinal cord of PXR^+/+^ and PXR^−/−^ mice after SCI (*n* = 6 per group). (E,G) Immunofluorescent staining and corresponding quantitative analysis showed the 4‐HNE expression in the spinal cord of PXR^+/+^ and PXR^−/−^ mice after SCI (*n* = 6 per group); * *p* ˂ 0.05, **p ˂ 0.01, ***p ˂ 0.001. Data were presented as mean ± SEM.

Since activation of PXR has been found to increase oxidative stress during hemorrhagic shock‐induced liver injury in mice,^49^ we speculated whether the protective effect of PXR knockout on SCI was related to oxidative stress. We first examined the activities of antioxidant enzymes, SOD and GPx, and the expression of three common chemical markers of oxidative stress, including MDA, 4‐HNE, and 8‐OXO. Compared to Sham groups, SCI groups showed a significant elevation of MDA content and decreased activity of SOD and GPx in spinal tissues. SCI/PXR^−/−^ group showed down‐regulated MDA content and up‐regulated activities of SOD and GPx (Figure 5A–C). The immunofluorescence signals of 4‐HNE and 8‐OXO were increased in SCI/PXR^+/+^ group, which were markedly reversed in SCI/PXR^−/−^ group (Figure 5D–G). Collectively, these findings demonstrated that PXR deficiency can alleviate oxidative stress after SCI.

### 3.6 Activation of PXR not only inhibits the functional recovery but also aggravates apoptosis and oxidative stress

**FIGURE 6:**
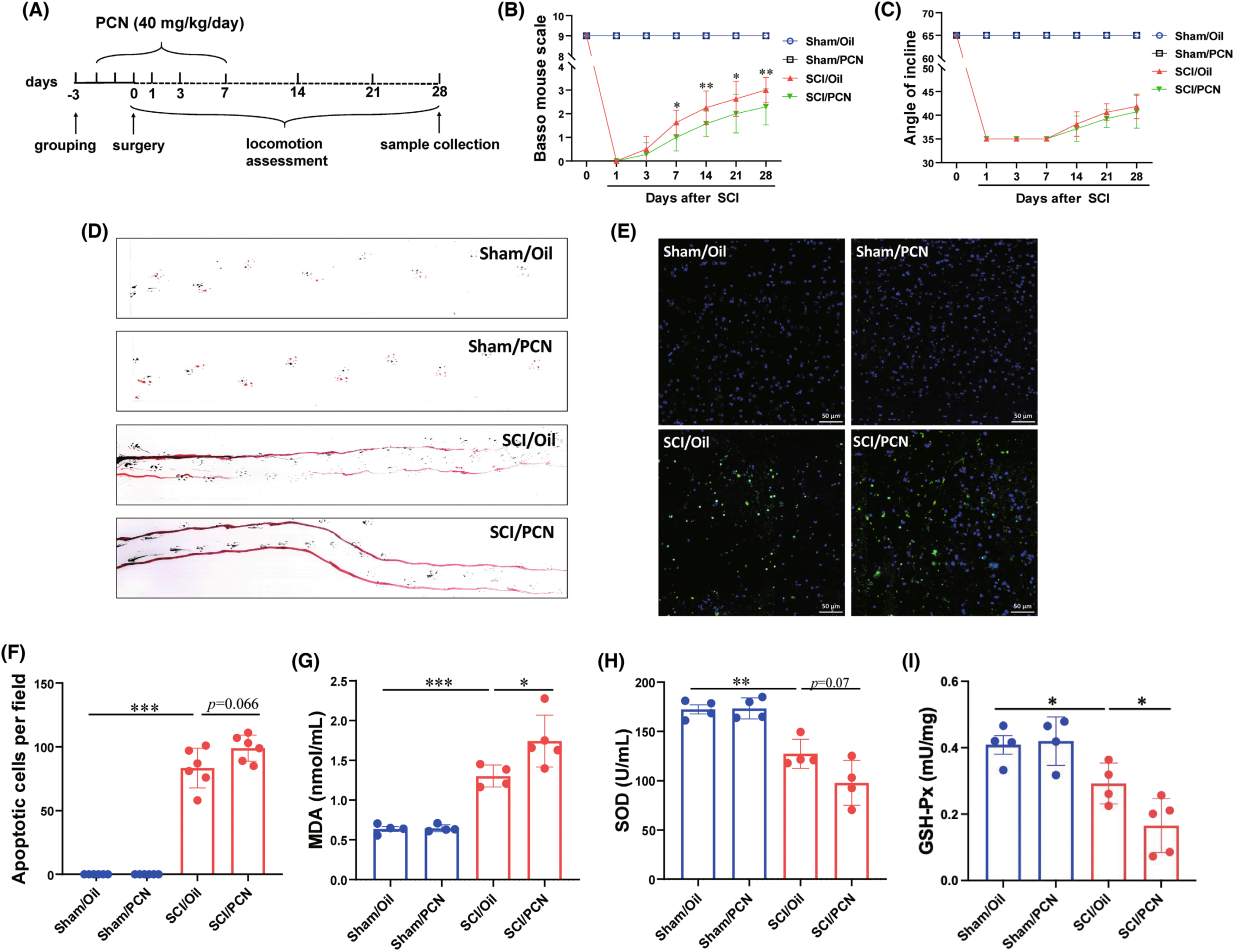
PXR activation aggravates spinal cord injury in mice. (A) Schematic procedure of PXR activation and SCI model construction. (B,C) Quantitative analysis of gross voluntary movement of wild‐type mice with PXR activation measured by BMS scoring analysis (B) and inclined plane test (C) at different stages after SCI (*n* = 8 per group). (D) Representative footprint images of wild‐type mice with PXR activation after SCI. (E) Apoptotic cells were detected by TUNEL staining after SCI in wild‐type mice with PXR activation. (F) Quantitative analysis of apoptosis in (E) (*n* = 6 per group). (G–I) Biochemical indexes, MDA content (G), SOD activity (H), and GPx activity (I), were measured in the spinal cord tissues of wild‐type mice with PXR activation after SCI (*n* = 6 per group); * *p* ˂ 0.05, **p ˂ 0.01 compared with 0 d in (B). * *p* ˂ 0.05, **p ˂ 0.01, ***p ˂ 0.001 in (F–I). Data were presented as mean ± SEM.

To determine the effect of PXR activation on SCI. We divided the male wild‐type C57BL/6 mice into four groups (*n* = 6–8 per group): Sham/Oil, Sham/PCN, SCI/Oil, and SCI/PCN. Mice were pretreated with PCN for 3 days to activate PXR before SCI and were administered daily the first 7 days after SCI as shown in the schematic diagram (Figure 6A). In the spinal cord, PCN could significantly increase the mRNA and protein levels of PXR (Figure S7). Mice subjected to SCI showed a significant decline in motor function, as shown by BMS scores (Figure 6B), inclined plane test (Figure 6C), and footprint behavioral assays (Figure 6D). The behavioral recovery was significantly reduced in SCI/PCN group compared with SCI/Oil group in BMS scores and footprint behavioral assays (Figure 6B,D). However, there was no difference in inclined plane test between SCI/Oil group and SCI/PCN group, probably due to the damage of hind limbs were too severe (Figure 6C). TUNEL staining was conducted for investigating the apoptosis in all groups. Compared with the Sham groups, a remarkedly elevated in the number of TUNEL‐positive cells in SCI groups. SCI/PCN group increased the number of apoptotic cells compared with SCI/Oil group (Figure 6E,F). Since the knockout of PXR alleviated oxidative stress after SCI, we further detected whether activation of PXR increased oxidative stress. Upon SCI, MDA content significantly increased, meanwhile, SOD and GPx activities were decreased, which were further aggravated in SCI/PCN group (Figure 6G–I). Together, these results indicated that PXR activation aggravated motor function damage, apoptosis, and oxidative stress after SCI.

### 3.7 The effect of PXR on SCI is related to the modulation of NRF2/HO‐1 pathway

**FIGURE 7:**
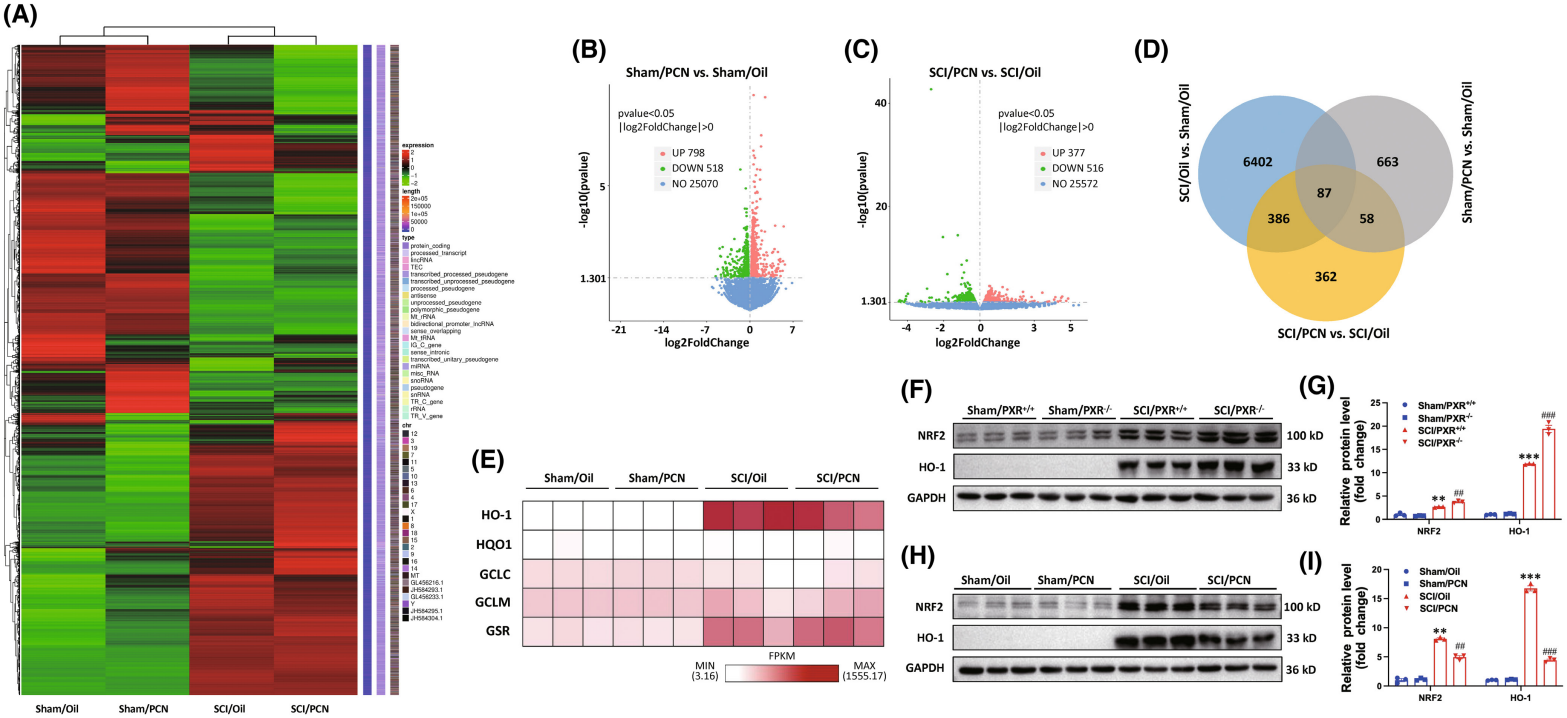
RNA‐Seq analysis of gene expression profile after PXR activation in mice with SCI. (A) Hierarchical clustering analysis was performed to compare the gene expression alteration in different groups; the red and green colors indicated respectively the genes upregulated and downregulated. (B) Volcano plots inferred the overall distribution of differentially expressed genes between Sham/PCN and Sham/Oil groups. (C) Volcano plots inferred the overall distribution of differentially expressed genes between SCI/PCN and SCI/Oil groups. (D) Venn diagram showed the genes unique to each group and shared among the three groups (SCI/Oil vs. Sham/Oil, Sham/PCN vs. Sham/Oil, and SCI/PCN vs. SCI/Oil); the gene number for each component was listed. (E) Heat map demonstrating hierarchical clustering of HO‐1, NQO‐1, GCLC, GCLM, and GSR, in which HO‐1 showed various expression patterns in different groups. (F) Western blot analysis showed the protein level of NRF2 and HO‐1 in the spinal cord of PXR^+/+^ and PXR^−/−^ mice after SCI. (G) Quantitative analysis of protein level in (F) (*n* = 3 per group). (H) Western blot analysis showed the protein level of NRF2 and HO‐1 in the spinal cord of wild‐type mice with PXR activation after SCI. (I) Quantitative analysis of protein level in (H) (*n* = 3 per group). **p ˂ 0.01, ***p ˂ 0.001 compared with Sham/PXR^+/+^; ^##^ *p* ˂ 0.01, ^###^ *p* ˂ 0.001 compared with SCI/PXR^+/+^ in (G). **p ˂ 0.01, ***p ˂ 0.001 compared with Sham/Oil; ^##^ *p* ˂ 0.01, ^###^ *p* ˂ 0.001 compared with SCI/Oil in (I). Data were presented as mean ± SEM.

To further understand and characterize the mechanisms by which PXR aggravates SCI, the spinal tissues (T9, 1 cm) taken from the central area of the injury on the 3 d after SCI were sampled and used to analyze transcriptomes of Sham/Oil, Sham/PCN, SCI/Oil, and SCI/PCN (*n* = 3 per group). The differentially expressed genes (DEGs) among the four groups were shown in the cluster heatmap (Figure 7A). In Figure 7B, the volcano plots of Sham/PCN and Sham/Oil group DEGs showed that 798 genes were up‐regulated and 518 genes were down‐regulated between the two groups. We further performed GO enrichment pathway analysis between these two groups and found that respiratory chain‐related, energy metabolism‐related, and oxidative stress‐related pathways were enriched (Figure S8). In Figure 7C, the volcano plots of SCI/PCN and SCI/Oil group DEGs showed that 377 genes were up‐regulated and 516 genes were down‐regulated between the two groups. The DEGs unique to SCI/Oil versus Sham/Oil, Sham/PCN versus Sham/Oil, and SCI/PCN versus SCI/Oil, and shared among these comparing groups were illustrated in the Venn diagram (Figure 7D). Nuclear factor erythroid 2‐related factor 2 (NRF2), a main sensor regulating the redox reaction, controls the expression of antioxidants, and detoxification enzymes including heme oxygenase‐1 (HO‐1), NAD(P)H dehydrogenase (quinone) 1 (NQO‐1), glutamate cysteine ligase catalytic subunit (GCLC), glutamyl cysteine ligase modulatory subunit (GCLM), and glutathione reductase (GSR). Therefore, we focused on these enzymes and analyzed their mRNA expression in our RNA‐seq data (Figure 7E). We found that the mRNA level of HO‐1 was remarkably increased after SCI and decreased with PCN treatment (Figure 7E). It has been reported that HO‐1 protein expression is low in most mammalian tissues and can be significantly up‐regulated by infection, ultraviolet irradiation, and oxidative stimuli.^50^, ^51^, ^52^, ^53^ Numerous studies have shown that HO‐1 activation in the CNS including AD, PD, and traumatic brain injury (TBI), has a strong protective effect on antioxidant, anti‐inflammation, and anti‐apoptosis.^54^, ^55^, ^56^, ^57^ We then assessed the protein level of NRF2 and HO‐1 in spinal cord tissues. The results showed that SCI increased the expression of NRF2 and HO‐1, which was further enhanced in SCI/PXR^−/−^ group (Figure 7F,G) and suppressed in SCI/PCN group (Figure 7H,I). Collectively, these results indicated that the NRF2/HO‐1 pathway may be related to regulating the effect of PXR on SCI.

### 3.8 PXR siRNA attenuates H_2_O_2_ ‐induced cell injury partially by activating NRF2/HO‐1 pathway in N2a

We further simulated the oxidative stress damage environment in vitro. Since PXR is mainly expressed in neurons (Figure 1E‐G), we investigated the effect of PXR on neuronal oxidative stress in a N2a H_2_O_2_‐induced injury model simulating the pathological process of SCI in vitro. Following treatment of the N2a with different concentrations of H_2_O_2_ for 24 h, CCK‐8 results showed that the cell survival rate decreased in a concentration‐dependent manner (Figure S9). 400 μM H_2_O_2_ was selected to induce cell damage in subsequent experiments. We knocked down the expression of PXR by siRNA in N2a cells. Western blot analysis was used to confirm that the expression of PXR was successively suppressed by transfection with siPXR compared with control siRNA (Figure S10). To verify the involvement of the NRF2/HO‐1 pathway in the neuroprotective effect of PXR siRNA on H_2_O_2_‐induced cell injury, an NRF2 inhibitor (ML385) was used.

**FIGURE 8:**
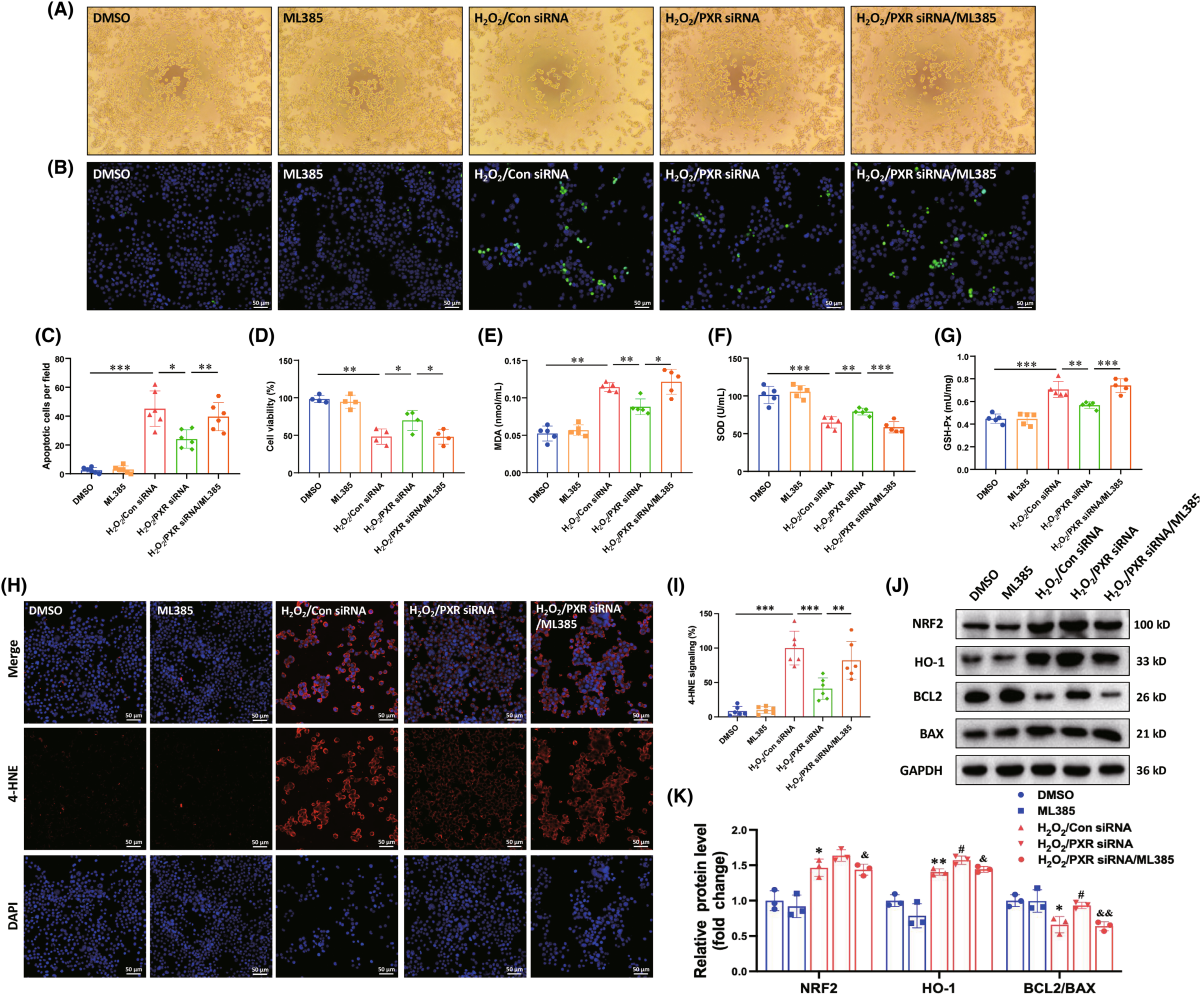
PXR knockdown protects neuronal damage induced by H_2_O_2_ in vitro. (A) Morphology and status of the cells treated with H_2_O_2_ after PXR knockdown with or without ML385 treatment. (B) Apoptosis was detected by TUNEL staining in the cells treated with H_2_O_2_ after PXR knockdown with/without ML385 treatment. (C) Quantitative analysis of apoptosis in (B) (*n* = 6 per group). (D) Cell viability was evaluated by CCK‐8 analysis in the cells treated with H_2_O_2_ after PXR knockdown with/without ML385 treatment. (E–G) Biochemical indexes, MDA content (E), SOD activity (F), and GPx activity (G), were measured in the cells treated with H_2_O_2_ after PXR knockdown with/without ML385 treatment; (H,I) Immunofluorescent staining and corresponding quantitative analysis showed the 4‐HNE expression in the cells treated with H_2_O_2_ after PXR knockdown with/without ML385 treatment (*n* = 6 per group). (J) Western blot analysis showed the protein level of NRF2, HO‐1, BCL2, and BAX in the cells treated with H_2_O_2_ after PXR knockdown with/without ML385 treatment. (K) Quantitative analysis of protein level in (J) (*n* = 3 per group). *p ˂ 0.05, **p ˂ 0.01, ***p ˂ 0.001 in (C–G, I). *p ˂ 0.05, **p ˂ 0.01 compared with DMSO, ^#^ *p* ˂ 0.05 compared with H_2_O_2_/Con siRNA, and ^&^ *p* ˂ 0.05, ^&&^ *p* ˂ 0.01 compared with H_2_O_2_/PXR siRNA in (K). Data were presented as mean ± SEM.

Cell survival significantly decreased after treatment with 400 μM H_2_O_2_ for 24 h, which was increased by the transfection with siPXR. However, the protective effect of PXR siRNA on cell survival was inhibited by ML385 (Figure 8A–D). As compared to control siRNA, the cells transfected with siPXR showed reduced apoptosis after H_2_O_2_ treatment. However, together treated with ML385, the anti‐apoptotic effect of PXR siRNA significantly decreased (Figure 8B,C). Furthermore, we examined oxidative stress biomarkers, MDA content, the immunofluorescence of 4‐HNE, and activity enzymes associated with anti‐oxidative stress, SOD and GPx, after H_2_O_2_ exposure. H_2_O_2_ remarkably raised the content of MDA and the fluorescence intensity of 4‐HNE, and the activity of SOD was significantly decreased, while transfection with siPXR weakened the upregulation of MDA, GPx and 4‐HNE level, and recovered the activity of SOD elicited by H_2_O_2_, which all been reversed by co‐treated with ML385 (Figure 8E–I). Unexpectedly, the GPx activity was increased after H_2_O_2_ treatment. This is not consistent with the tissue level trend, and we speculated that it may be attributed to the injury which is within an adaptive range and causes the increase of the GPx activity reactivity. Moreover, the western blot analysis (as determined by assaying the levels of NRF2, HO‐1, BAX, and BCL2) showed that PXR siRNA can activate the NRF2/HO‐1 pathway and diminish apoptosis, which was reversed by ML385 (Figure 8J,K). Therefore, blockage of NRF2/HO‐1 pathway by ML385 abolished the neuroprotective effect of PXR siRNA on the H_2_O_2_‐induced oxidative stress response and apoptosis in N2a cells. The results demonstrated that the knockdown of PXR could alleviate H_2_O_2_‐induced oxidative stress response and apoptosis in N2a cells by activating NRF2/HO‐1 signaling pathway.

### 3.9 PXR activation aggravates H_2_O_2_ ‐induced cell injury partially by inhibiting NRF2/HO‐1 pathway in N2a

**FIGURE 9:**
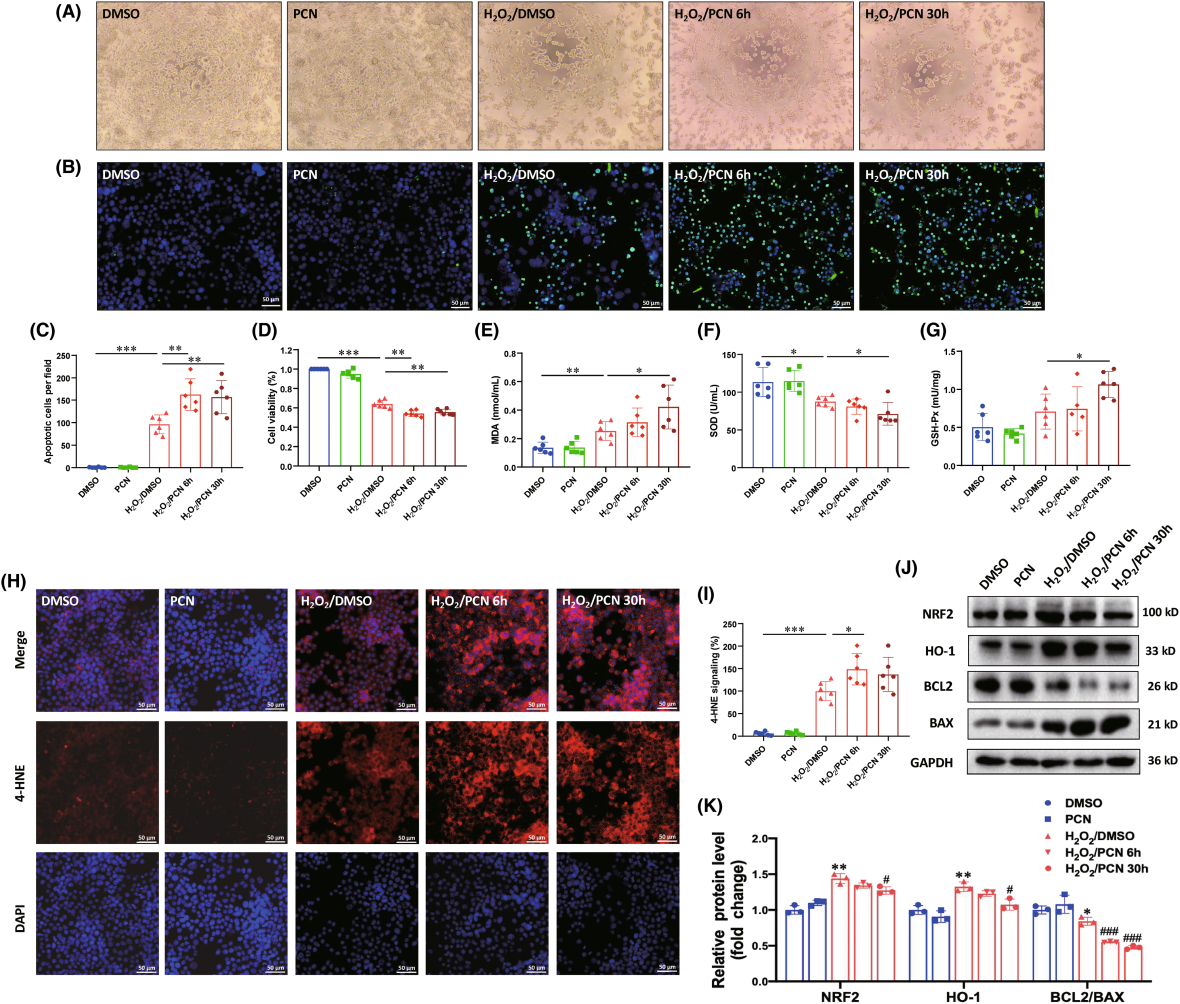
PXR activation aggravates neuronal damage induced by H2O2 in vitro. (A) Morphology and status of the cells treated with H_2_O_2_ after PXR was activated by PCN. (B) Apoptosis was detected by TUNEL staining in the cells treated with H_2_O_2_ after PXR was activated by PCN. (C) Quantitative analysis of apoptosis in (B) (*n* = 6 per group). (D) Cell viability was evaluated by CCK‐8 analysis in the cells treated with H_2_O_2_ after PXR was activated by PCN. (E–G) Biochemical indexes, MDA content (E), SOD activity (F), and GPx activity (G), were measured in the cells treated with H_2_O_2_ after PXR was activated by PCN. (H,I) Immunofluorescent staining and corresponding quantitative analysis showed the 4‐HNE expression in the cells treated with H_2_O_2_ after PXR was activated by PCN (*n* = 6 per group). (J) Western blot analysis showed the protein level of NRF2, HO‐1, BCL2, and BAX in the cells treated with H_2_O_2_ after PXR was activated by PCN. (K) Quantitative analysis of protein level in (J) (*n* = 3 per group). *p ˂ 0.05, **p ˂ 0.01, ***p ˂ 0.001 in (C–G, I). *p ˂ 0.05, **p ˂ 0.01 compared with DMSO; ^#^ *p* ˂ 0.05, ^###^ *p* ˂ 0.001 compared with H_2_O_2_/DMSO in (K). Data were presented as mean ± SEM.

To investigate the influence of PXR activation on H_2_O_2_‐induced N2a cells injury, we treated N2a cells with PCN and found that the mRNA level and protein level of PXR reached the maximum at 6 h after 40 μM PCN treatment, thus, in the subsequent experiment, we pretreated with PCN for 6 h (Figure S11). The cell number in PCN pretreatment group (H_2_O_2_/PCN 6 h) was significantly lower than that in DMSO pretreatment group (H_2_O_2_/DMSO) after H_2_O_2_ treatment. Moreover, PCN pretreatment followed by H_2_O_2_ exposure and continued PCN stimulation (H_2_O_2_/PCN 30 h group) further aggravated neuronal death (Figure 9A–D). We tested the MDA content, the fluorescence intensity of 4‐HNE, and the activities of SOD and GPx. The MDA content, the fluorescence intensity of 4‐HNE and the activity of GPx in H_2_O_2_/PCN 6 h group and H_2_O_2_/PCN 30 h group were higher than that in the H_2_O_2_/DMSO group. The activity of SOD showed the opposite trend that H_2_O_2_/PCN 6 h group and H_2_O_2_/PCN 30 h group had significantly lower activity of SOD compared to the H_2_O_2_/DMSO group (Figure 9E–I). To confirm whether PXR aggravated oxidative stress by inhibiting NRF2/HO‐1 signaling pathway after SCI, we examined the expression of NRF2 and HO‐1 by western blot analysis, which showed that PCN had an inhibitory effect on NRF2 and HO‐1 after H_2_O_2_ exposure (Figure 9J,K). Meanwhile, PCN treatment aggravated the decrease in BCL2 and the increase of BAX regulators of apoptosis after H_2_O_2_ exposure (Figure 9J,K). Overall, the results showed that activation of PXR aggravated oxidative stress response and apoptosis in part partly through inhibition of NRF2/HO‐1 signaling pathway in H_2_O_2_‐induced N2a cells.

## 4 DISCUSSION

The present study demonstrates the protective effect of PXR deficiency on SCI recovery and proposes a working mechanism. In the clip‐compressive SCI model, PXR gene‐knockout (PXR^−/−^) mice improved the behavioral recovery of motor function, suppressed oxidative stress, apoptosis, and inflammation after SCI. Further, mice with PXR activation showed worse motor function recovery and increased oxidative stress and apoptosis after SCI. Moreover, for in vitro study, PXR siRNA alleviated oxidative stress and apoptosis stimulated by H_2_O_2_ in N2a mouse neuroblast cells, whereas the opposite effects occurred after PXR stimulation. Mechanistically, we found that PXR has a negative effect on SCI and aggregates SCI recovery by inhibiting NRF2/HO‐1 signaling pathway.

As a devastating condition, acute traumatic SCI leads to substantial permanent morbidity. The primary injury is prominently featured in the lesion epicenter, followed by the reversible secondary injury including the changes in inflammatory response, oxidative stress, neuronal apoptosis, and glial scar formation. Thus, the secondary injury of SCI has become the mainstream direction of SCI research. Currently, methylprednisolone (MP) is the only FDA‐approved drug in treating SCI as its anti‐inflammatory effect.^58^ However, due to the numerous side effects of femoral head necrosis, infections, and bleeding, the application of MP is controversial and has no longer been recommended for routine use for treating SCI.^59^, ^60^, ^61^ There are even recent studies showing that the application of MP is ineffective in patients with SCI.^62^, ^63^ Therefore, it is urgent to provide new promising molecular therapeutic targets and elucidate their mechanism for SCI treatment.

Multiple lines of evidence have shown that NRs play an important role in SCI. Liver X receptor (LXR) agonist treatment significantly reduced the SCI‐induced tissue damage and improved the motor function in CD1 mice.^64^ It was initially shown that the total amount of retinoid X receptor α (RXRα) in the central lesion decreased at 4, 7, and 14 days after SCI,^65^ and a recent study found that the downregulation of RXRα can promote the regeneration after SCI by targeting p66shc.^21^ The agonist of peroxisome proliferator‐activated receptor α (PPARα) reduced inflammation and promoted recovery of function after SCI.^66^, ^67^ However, up to now, the role of PXR in the SCI has not been studied. Our study found that PXR is widely expressed in the CNS of mice, with the highest expression in the hypothalamus, followed by the cerebral cortex and spinal cord, and for the first time in the spinal cord, PXR‐specific expression was found in neurons, but not in astrocytes and microglia (Figure 1). Further, we found that the expression of PXR was decreased after SCI with the lowest expression on the third day after SCI, and then gradually returned to normal level (Figure 2). The previous reports showed that inflammation and oxidative stress may suppress the expression of PXR.^68^, ^69^, ^70^ The inflammatory reactions and oxidative stress reactions mainly take place within a week after SCI. We suspect that the expression of PXR decreased from the first day after SCI and reached the lowest level on the third day, which may be the result of the combined effect of inflammation and oxidative stress to inhibit the expression of PXR. Previous reports suggest that hemorrhagic shock has a dynamic effect on the expression of PXR, and the hemorrhagic shock‐responsive suppression of PXR may represent a protective response and/or secondary response to HS‐induced liver injury.^49^ Therefore, the reduction in PXR levels may also be a protective and/or a pathological response to SCI.

We further found that PXR deficiency promoted the recovery of motor function, reduced the area of injury site, apoptosis, inflammation, and oxidative stress after SCI (Figures 3 and 5). In contrast, pharmacological activation of PXR aggravated the damage after SCI (Figure 6). It is worth mentioning that our study demonstrated for the first time that PXR deficiency alleviated the inflammatory response after SCI, whereas previous reports have suggested that pharmacological activation of PXR has anti‐inflammatory effects in inflammatory bowel disease through inhabiting NF‐kappaB signaling.^71^ This suggests that PXR may play diametrically opposite roles in different systems.

To further investigate the molecular mechanism of how PXR regulates the development of SCI, transcriptome mRNA sequencing was performed which revealed that PXR activation significantly influenced the expression level of HO‐1, a classic downstream target of NRF2, in the spinal cord tissues after SCI. We further verified that PXR modulated NRF2/HO‐1 pathway at the protein level in vivo and in vitro (Figures 7 and 9). NRF2, a main sensor regulating the redox reaction, controls the expression of antioxidants and detoxification enzymes including HO‐1.^72^, ^73^ Recent studies have shown that activation of NRF2/HO‐1 signal inhibits oxidative stress, apoptosis, inflammation, and promotes functional recovery after SCI.^73^, ^74^, ^75^ Previous studies have shown that PXR activation potentiates ritonavir hepatotoxicity, which involved in oxidative stress and endoplasmic reticulum stress through CYP3A4‐dependent pathways.^31^ Activation of PXR increased oxidative stress in the mice to hemorrhagic shock‐induced liver injury,^49^ and sensitized colon cancer LS180 cells to an oxidative toxicant.^76^ In addition, PXR promoted oxidative stress in human neural (SH‐SY5Y) and glial (U373‐MG) cell lines induced by organophosphorus pesticide monocrotophos (MCP).^77^ The spinal cord is particularly sensitive to free‐radical‐mediated injury due to its high rate of oxidative metabolic activity and high lipid content. Following SCI, a large number of free radicals are produced, which in turn induce an oxidative stress response, is the key event of secondary injury.^78^, ^79^ Increasing evidence indicates that effective inhibition of oxidative stress may be a suitable strategy for the treatment of SCI.^80^, ^81^, ^82^ In our study, the oxidative stress response was suppressed in PXR^−/−^ mice and H_2_O_2_‐stimulated N2a cells treated with siPXR, accompanied by a significant reduction in oxidative stress markers, such as MDA, 4‐HNE, 8‐OXO, and the elevation activity of anti‐oxidants, SOD and GPx, at the same time. In contrast, pharmacological activation of PXR aggravated oxidative stress both in vivo and in vitro.

However, the present study has certain limitations. First, the PXR^−/−^ mice used in the study were a whole‐body knockout, not specifically knockout in the spinal cord, so the effect of PXR knockout in other systems on SCI cannot be excluded. Secondly, the exact mode of how PXR inhibits NRF2/HO‐1 is still unclear, further studies are needed to investigate the mechanism. Moreover, since the expression of PXR is much higher in the liver and intestine than in the spinal cord, the side effects should be considered when using PXR antagonist in clinical practice. Therefore, tissue‐ and organ‐specific inhibitors for PXR are required to be urgently exploited and utilized.

In summary, our study not only revealed the deleterious role of PXR in SCI, but also implicated that PXR prevented SCI recovery by inhibiting NRF2/HO‐1 signaling. Therefore, inhibition of PXR may serve as a potential target for treatment after SCI. Meanwhile, since nearly 60% of commercial drugs can activate PXR to some extent, cautions should be paid on the application of PXR‐activating drugs.

## AUTHOR CONTRIBUTIONS

Conceptualization: Z‐L.L., L‐N.X., and J.Y.; methodology: L‐N.X., Z‐X.H., Z‐F.J., and C.Z.; software: Z‐L.L., L‐N.X., and X‐W.S.; validation: W‐H.M., H‐T.L., and W.L.; formal analysis: L‐J.S.; resources: Z‐F.J; data curation: S‐B.L. and G‐Y.W.; writing—original draft preparation: L‐N.X.; writing—review and editing: Z‐L.L. and J.Y.; funding acquisition: Z‐L.L. All authors have read and agreed to the published version of the manuscript.

## CONFLICT OF INTEREST STATEMENT

The authors declare no conflict of interest.

## Supporting information


Supplementary Files

## Data Availability

The data that support the findings of this study are available from the corresponding author upon reasonable request.
