# Supplementary material for: Pregnane X receptor (PXR) deficiency protects against spinal cord injury by activating NRF2/HO‐1 pathway
Source: CNS Neurosci Ther. 2023 Jun 2;29(11):3460–78. doi: 10.1111/cns.14279 (PMC10580351; doi:10.1111/cns.14279)
Supplement: Supplementary file 1 — Supplementary Files [file CNS-29-3460-s001.docx]

**
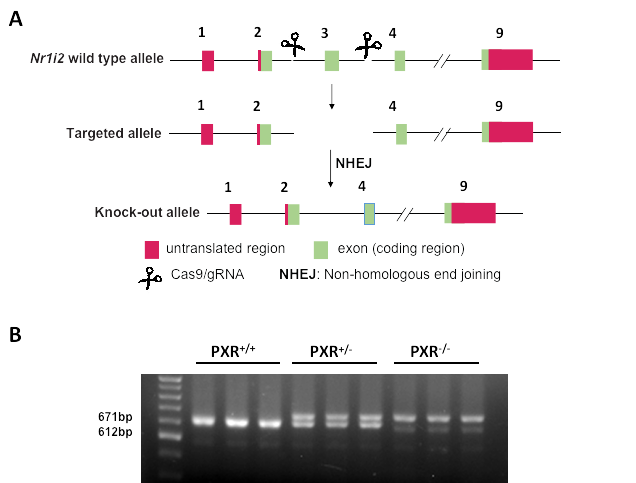
**

**Figure S1. Construction and genotyping of PXR gene-knockout mice.**

1. The schematic diagram indicated the construction for engineering the PXR gene-knockout mice; **(B)** Genotyping of the wild-type (PXR^+/+^) mice, heterozygous (PXR^+/-^) mice, and PXR gene-knockout (PXR^-/-^) mice. The genomic DNA extracted from mouse tail was used for genotyping by PCR. The PCR products of the tail DNA samples from PXR^+/+^, PXR^+/-^ and PXR^-/-^ mice include only the 612 bp fragment, both 612 bp and 671 bp fragments, and only the 671 bp fragment, respectively.
2.
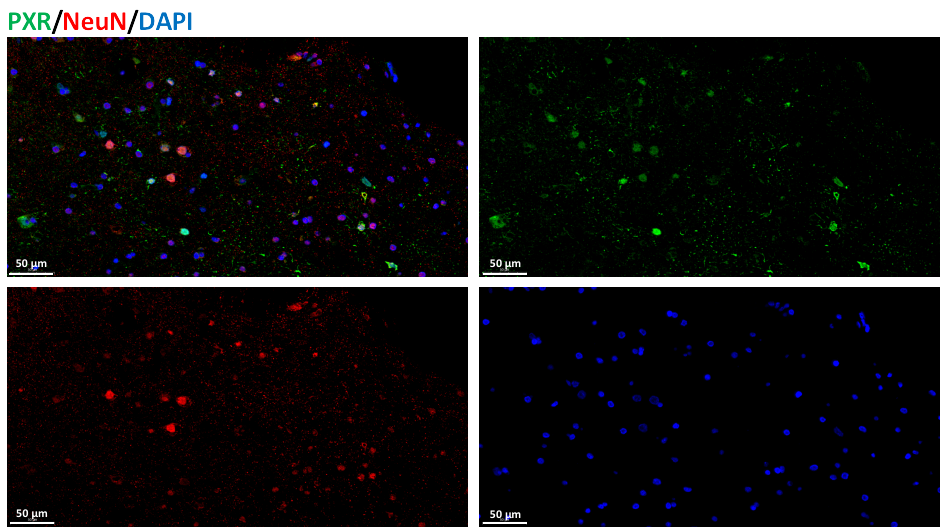


**Figure S2. PXR expression in a human brain specimen from a patient with intracranial hematoma**

**
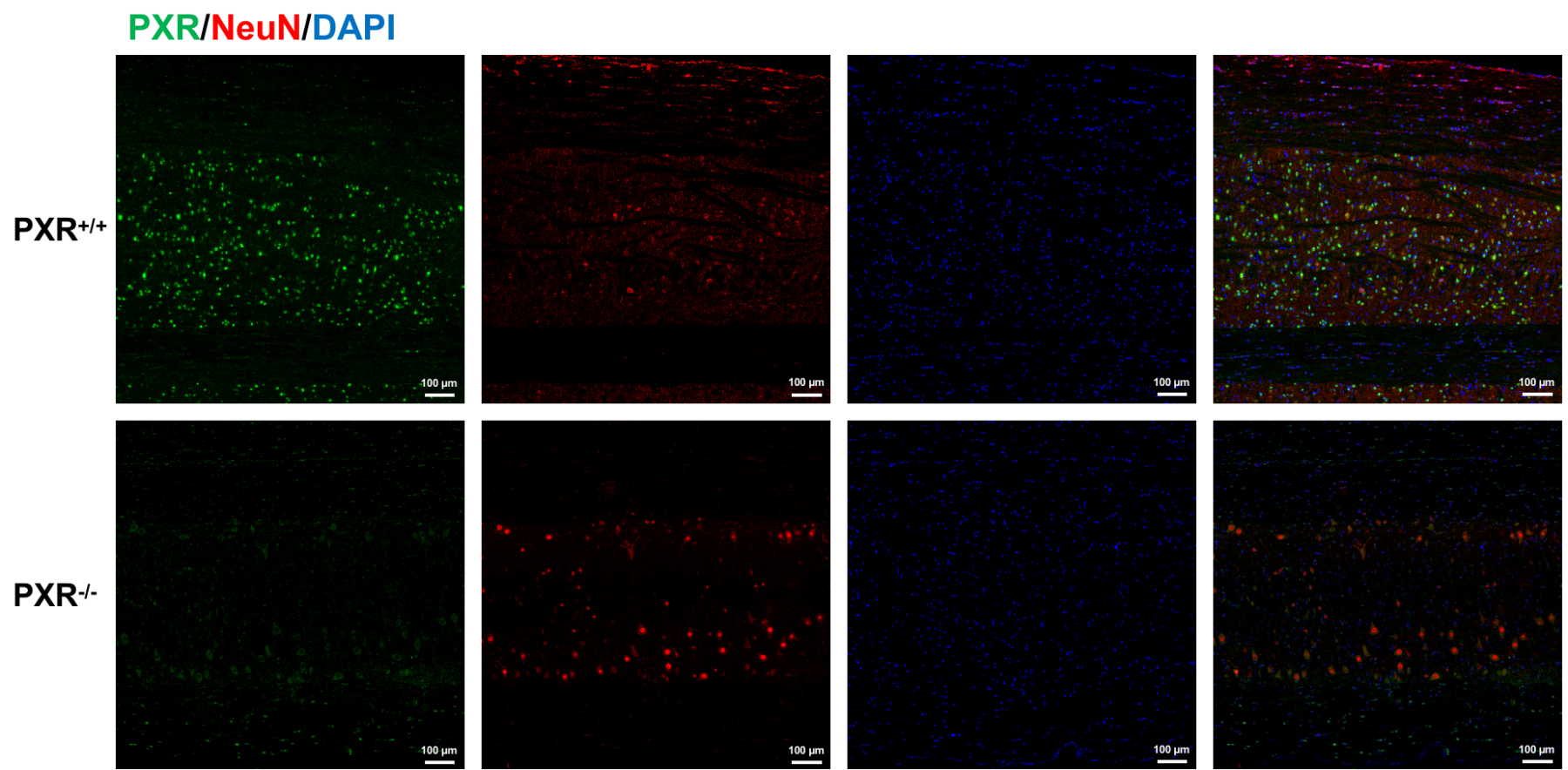
**

**Figure S3. PXR knockout validation in PXR^-/-^ mouse by immunofluorescence staining**


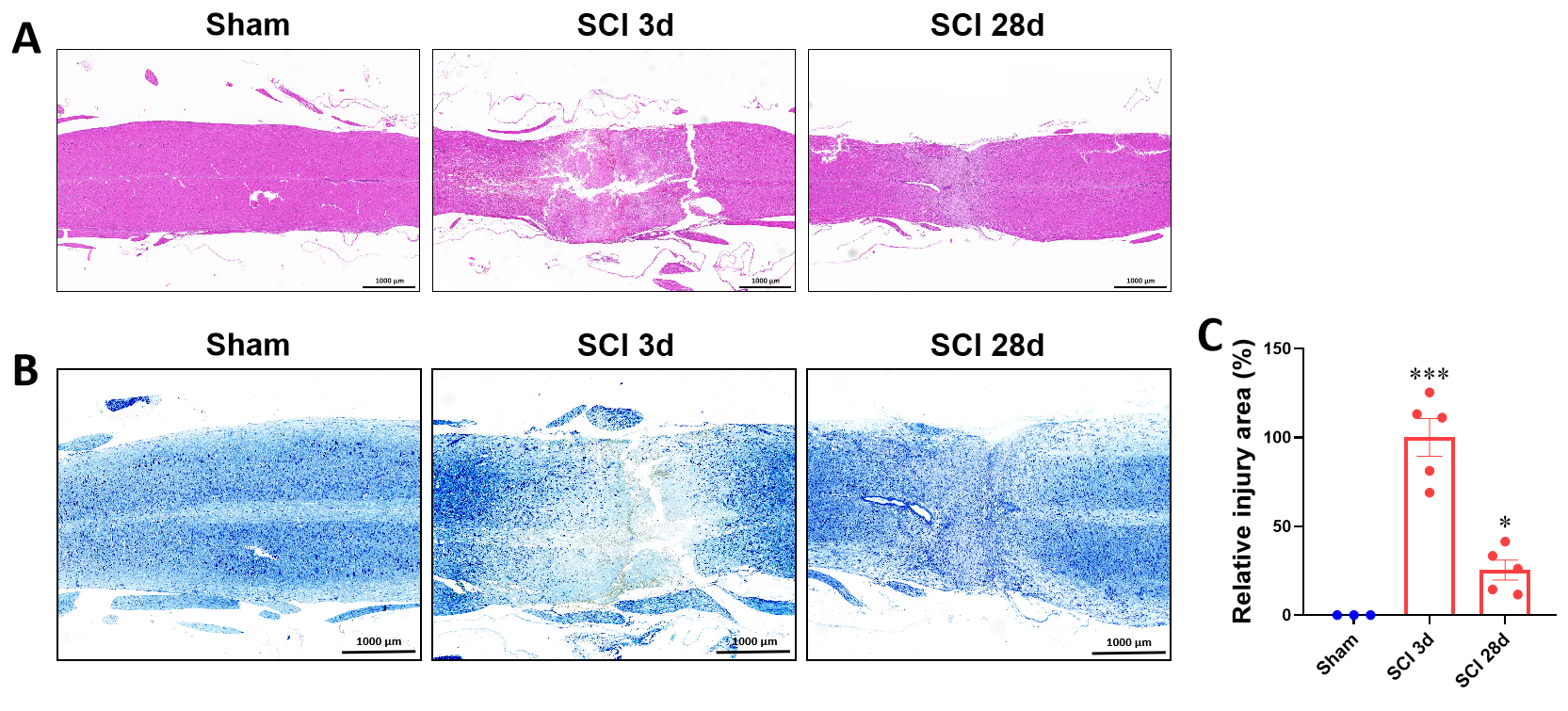


**Figure S4. Pathological feature after spinal cord injury**

1. HE staining images showed the pathological characteristics of SCI on day 3 and day 28; **(B)** Typical Nissl staining images showed the injury area in the spinal cords after SCI on day 3 and day 28; **(C)** Quantitative analysis of the injury area in the spinal cords as shown in **(B)**. **p*˂0.05, ****p* ˂0.001 compared with Sham. Data were presented as mean ± SEM.


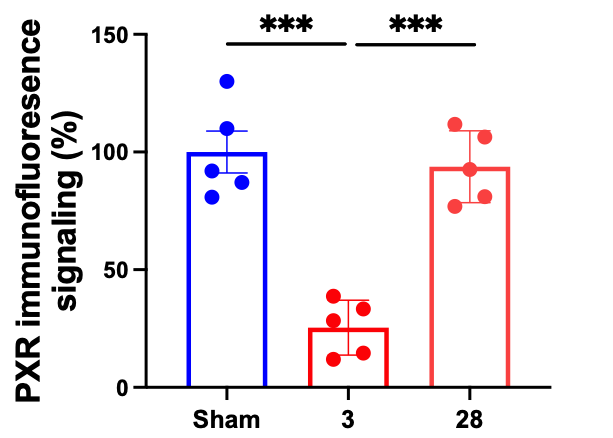


**Figure S5. Quantitative analysis of immunofluorescence staining in Figure 2G**


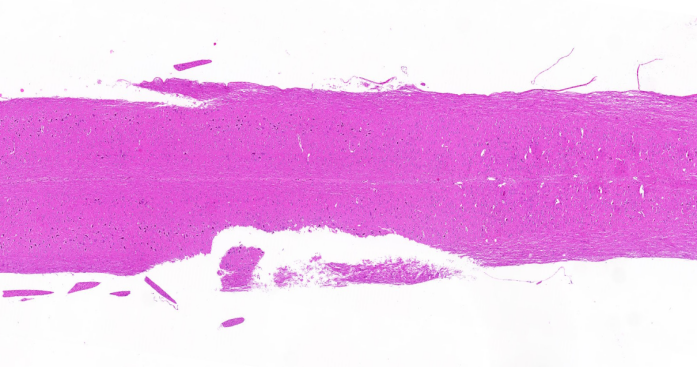


**1000 µm**

**Sham/PXR^+/+^**


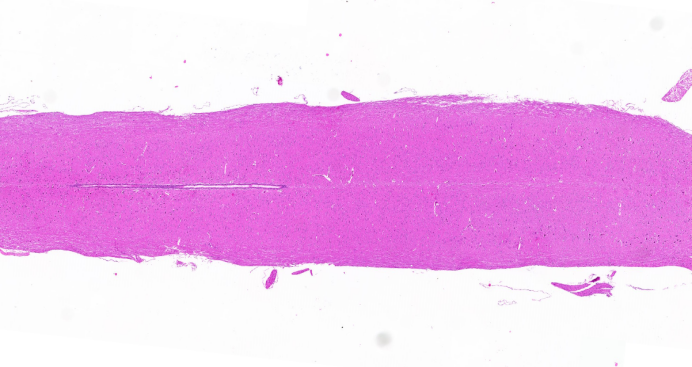


**1000 µm**

**Sham/PXR^-/-^**


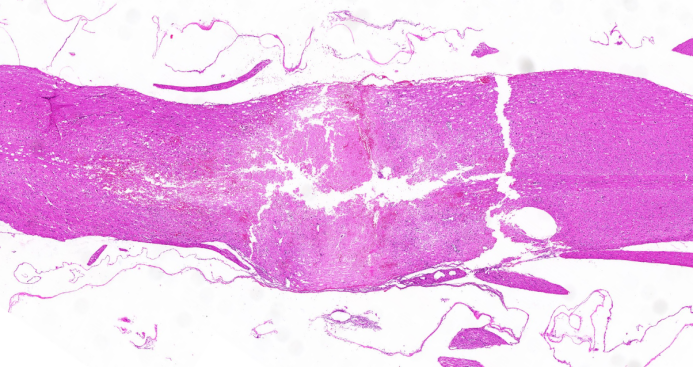


**1000 µm**

**SCI/PXR^+/+^**


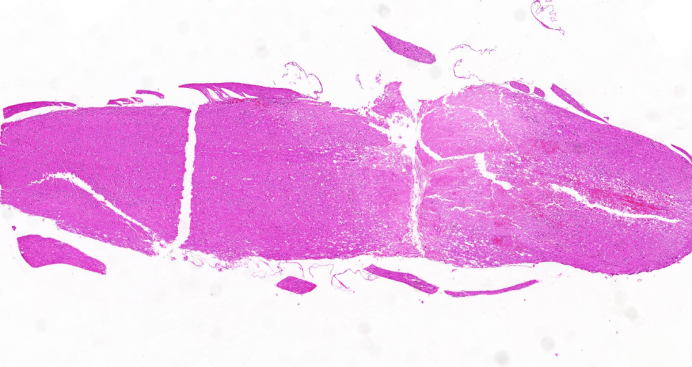


**1000 µm**

**SCI/PXR^-/-^**

**Figure S6. Pathological feature of PXR^-/-^ mice after spinal cord injury.**

HE staining showed the spinal cord damage of PXR^+/+^ and PXR^-/-^ mice after SCI compared with that of mice with sham operation.

**
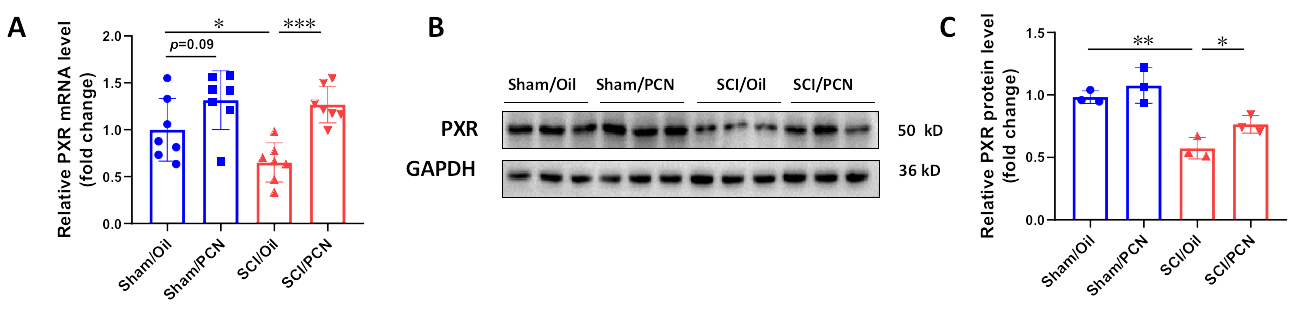
**

**Figure S7. PCN treatment activates PXR in mouse spinal cord**

1. Real-time PCR analysis showed the relative mRNA level of PXR after PCN treatment (n=7 per groups); **(B)** Western blot analysis showed the protein expression of PXR after PCN treatment; **(C)** Quantitative analysis of PXR protein level as shown in **(B)** (n=3 per groups). **p*˂0.05, ***p* ˂0.01. Data were presented as mean ± SEM.

**
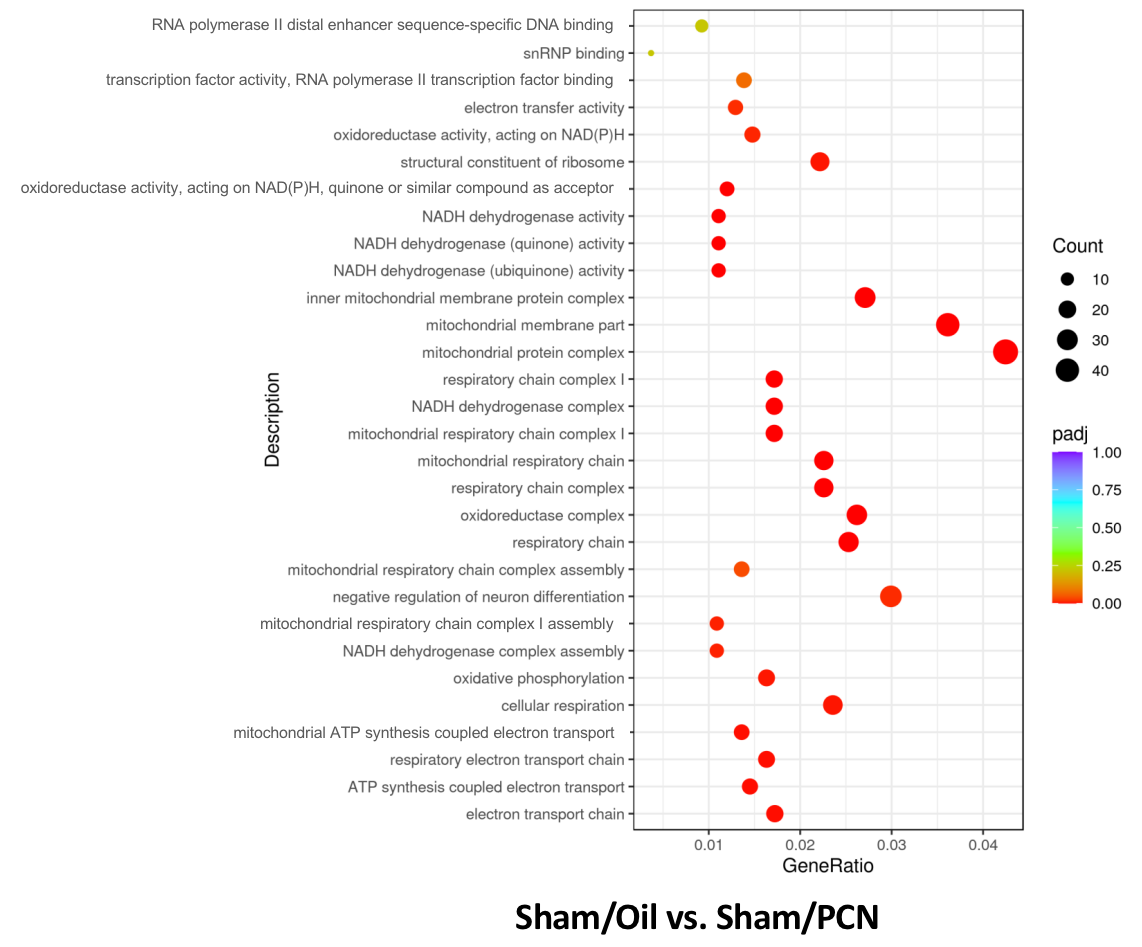
**

**Figure S8. GO enrichment analysis between Sham/PCN and Sham/Oil groups**

**
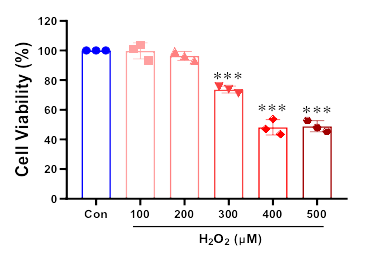
**

**Figure S9. Cell viability after H_2_O_2_ treatment at different concentration.**

****p* ˂0.001 compared with Con. Data were presented as mean ± SEM.

**
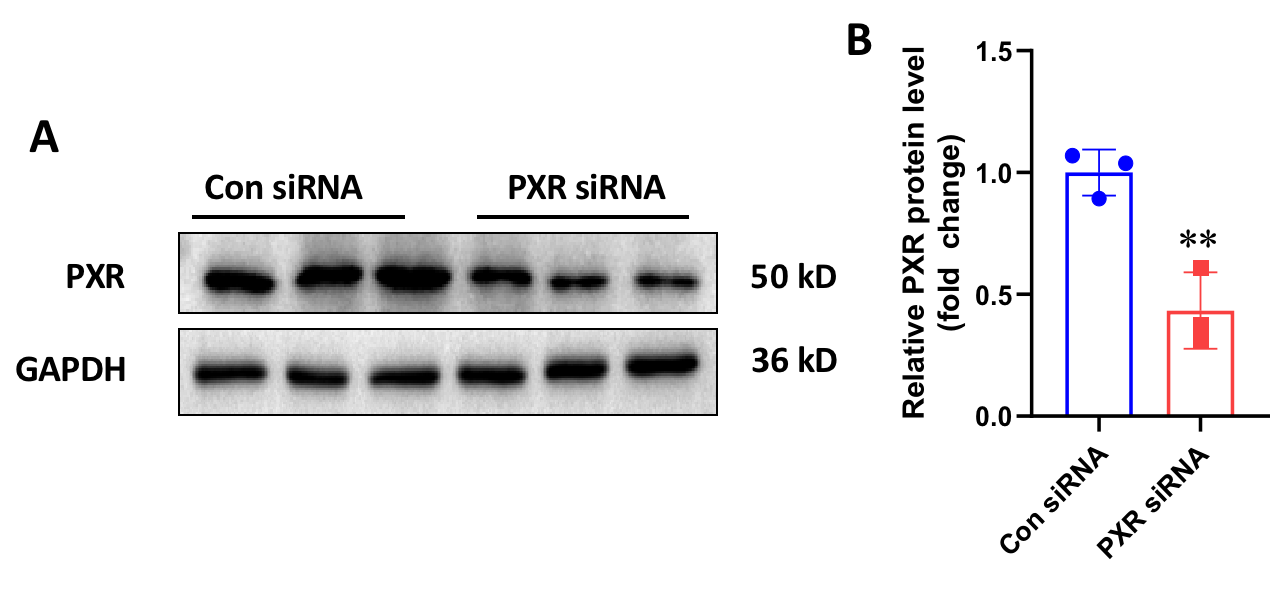
**

**Figure S10. Knockdown effect of PXR siRNA in N2a cells**

1. Western blot analysis showed the protein expression of PXR after PXR siRNA treatment; **(B)** Quantitative analysis of PXR protein level as shown in **(A)** (n=3 per groups). ***p* ˂0.01 compared with Con siRNA. Data were presented as mean ± SEM.

**
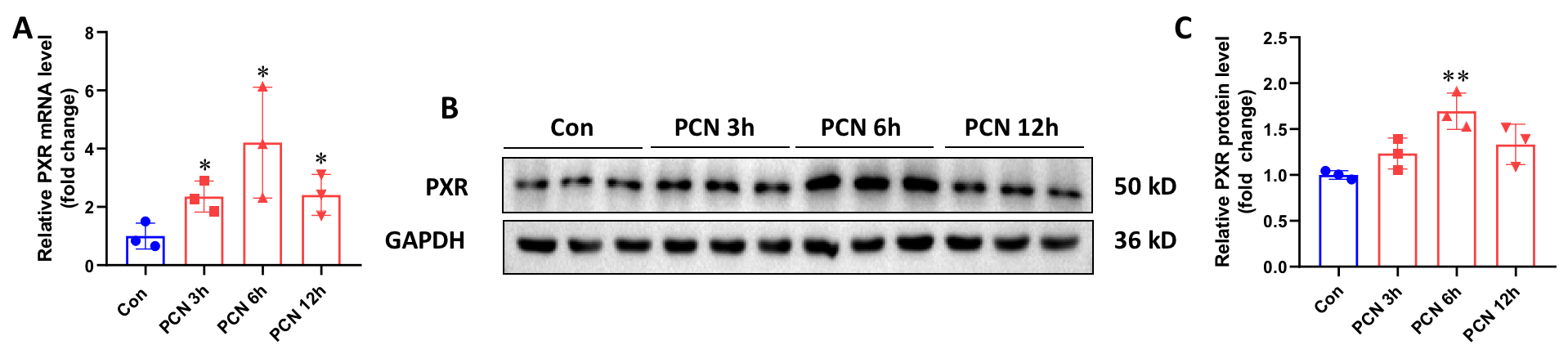
**

**Figure S11. PXR activation by PCN in N2a cell**

**(A)** Real-time PCR analysis showed the relative mRNA level of PXR after PCN treatment in N2a cells (n=3 per groups); **(B)** Western blot analysis showed the protein expression of PXR after PCN treatment in N2a cells; **(C)** Quantitative analysis of PXR protein level as shown in **(B)** (n=3 per groups). ***p* ˂0.01 compared with Con. Data were presented as mean ± SEM.
